# Whole genome identification, molecular docking and expression analysis of enzymes involved in the selenomethionine cycle in *Cardamine hupingshanensis*

**DOI:** 10.1186/s12870-024-04898-9

**Published:** 2024-03-19

**Authors:** Xixi Zeng, Guoqiang Luo, Zhucheng Fan, Zhijing Xiao, Yanke Lu, Qiang Xiao, Zhi Hou, Qiaoyu Tang, Yifeng Zhou

**Affiliations:** 1 Hubei Key Laboratory of Biological Resources Protection and Utilization, Enshi, China Enshi; 2 Hubei Key Laboratory of Selenium Resource Research and Biological Application, Enshi, China 44500; 3 College of Forestry and Horticulture, Hubei Minzu University, Enshi, China 44500; 4 College of Biological and Food Engineering, Hubei Minzu University, Enshi, China 44500; 5 Hubei Engineering Research Center of Selenium Food Nutrition and Health Intelligent Technology, Enshi, China 44500

**Keywords:** *Cardamine Hupingshanensis*, Selenomethionine cycle, Molecular docking, Gene expression, Selenium stress response

## Abstract

**Background:**

The selenomethionine cycle (SeMTC) is a crucial pathway for the metabolism of selenium. The basic bioinformatics and functions of four enzymes involved in the cycle including S-adenosyl-methionine synthase (MAT), SAM-dependent methyltransferase (MTase), S-adenosyl-homocysteine hydrolase (SAHH) and methionine synthase (MTR), have been extensively reported in many eukaryotes. The identification and functional analyses of SeMTC genes/proteins in *Cardamine hupingshanensis* and their response to selenium stress have not yet been reported.

**Results:**

In this study, 45 genes involved in SeMTC were identified in the *C. hupingshanensis* genome. Phylogenetic analysis showed that seven genes from *ChMAT* were clustered into four branches, twenty-seven genes from *ChCOMT* were clustered into two branches, four genes from *ChSAHH* were clustered into two branches, and seven genes from *ChMTR* were clustered into three branches. These genes were resided on 16 chromosomes. Gene structure and homologous protein modeling analysis illustrated that proteins in the same family are relatively conserved and have similar functions. Molecular docking showed that the affinity of SeMTC enzymes for selenium metabolites was higher than that for sulfur metabolites. The key active site residues identified for ChMAT were Ala^269^ and Lys^273^, while Leu^221/231^ and Gly^207/249^ were determined as the crucial residues for ChCOMT. For ChSAHH, the essential active site residues were found to be Asn^87^, Asp^139^ and Thr^206/207/208/325^. Ile^204^, Ser^111/329/377^, Asp^70/206/254^, and His^329/332/380^ were identified as the critical active site residues for ChMTR. In addition, the results of the expression levels of four enzymes under selenium stress revealed that *ChMAT3-1* genes were upregulated approximately 18-fold, *ChCOMT9-1* was upregulated approximately 38.7-fold, *ChSAHH1-2* was upregulated approximately 11.6-fold, and *ChMTR3-2* genes were upregulated approximately 28-fold. These verified that SeMTC enzymes were involved in response to selenium stress to varying degrees.

**Conclusions:**

The results of this research are instrumental for further functional investigation of SeMTC in* C. hupingshanensis*. This also lays a solid foundation for deeper investigations into the physiological and biochemical mechanisms underlying selenium metabolism in plants.

**Supplementary Information:**

The online version contains supplementary material available at 10.1186/s12870-024-04898-9.

## Introduction

As a trace and essential element for humans and animals, selenium is believed to be a beneficial element that promotes plant growth and takes part in other physiological processes [1]. Plants can be separated into three major categories regarding the ability to accumulate selenium: nonaccumulators (accumulating less than 100–1000 mg Se kg^−1^), secondary accumulators (accumulating 100–1000 mg Se kg^−1^), and hyperaccumulators (accumulating over 1000 mg Se kg^−1^ without any toxicity symptoms) [2]. *Neptunia amplexicaulis* (Fabaceae), *Cardamine hupingshanensis* (Brassicaceae), *Stanleya pinnata* (Brassicaceae) and *Astragalus bisulcatus* (Fabaceae) growing on seleniferous soils without any toxicity symptoms was considered to selenium hyperaccumulators [3, 4]. *N. amplexicaulis* is one of the strongest known Se hyperaccumulators on earth, with up to 13,600 mg Se kg^−1^ total in young leaves and an average concentration of 4334 mg Se kg^−1^ [5, 6]. The selenium content is averaging 2482 mg Se kg^−1^ in leaf of *S. pinnata* [7], and the selenium content in leaf of *A. bisulcate* is averaging 3045 mg Se kg^−1^ [8]. Selenium existed in the form of methyl-selenocysteine (MeSeCys) and selenomethionine (SeMet) in *N. amplexicaulis* and was found to mainly accumulate in the flowers, pods, young leaves, and taproots [9]. High concentrations of MeSeCys and SeMet were also shown to be in *A. bisulcate* and *S. pinnata* [10, 11]. As evidenced by existing studies, selenium has a pronounced effect on the growth of selenium hyperaccumulators including *N. amplexicaulis*, *S. pinnata*, and *A. bisulcate*, such as promoting the development of roots and limiting the uptake and accumulation of other heavy metals [12]. Selenium may activate the protective mechanisms involved in selenium hyperaccumulator oxidative stress by superoxide dismutase (SOD) and glutathione peroxidase (GPx) for example, the concentrations of glutathione and ascorbic acid were higher when *S. pinnata* was treated with 20 µM selenate [13]. Meanwhile, constitutively higher levels of hormones were observed in *S. pinnata*, including methyl jasmonate (MeJA), jasmonic acid (JA), salicylic acid (SA) and ethylene (ET), which play an important signaling role in selenium hyperaccumulation [13]. These hyperaccumulators are important for understanding the mechanism of selenium tolerance, detoxification, enrichment capabilities and metabolic pathways.

**Fig. 1:**
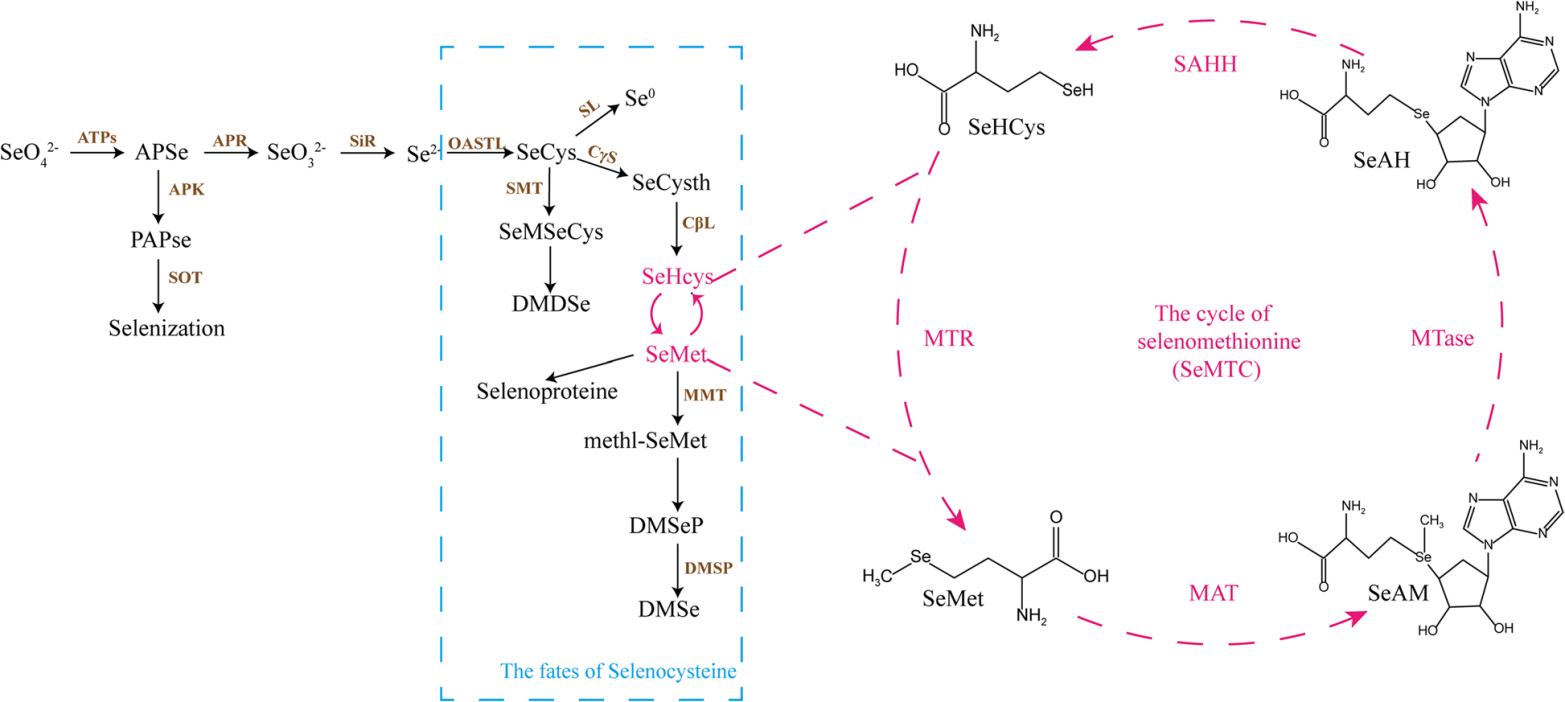
Schematic diagram of selenium metabolism and the cycle of selenomethionine in plants [17]. ATPs: ATP sulfurylase; APSe: adenosine 5’-phosphoselenate; APK: adenosine 5’-phosphosulfate kinase; PAPSe: phospho adenosine phosphor-selenate; SOT: sulfotransferase; APR: adenosine 5’-phosphosulfate reductase; SiR: sulfite reductase; OASTL: O-acetylserine (thiol) lyase; SeCys: selenocysteine; SMT: selenocysteine methyltransferase; SeMSeCys: selenomethylselenocysteine; DMDSe: dimethyl diselenide; SL: SeCyslyase; CγS: cystathionine gamma synthase; SeCysth: selenocystathionine; CβL: cystathionine beta lyase; SeHcys: selenium homocysteine; MMT: methionine methyl transferase; methl-SeMet: selenium methyl selenomethionine; DMSeP: dimethylselenonium propionate; DMSP: dimethylsulfoniopropionate lyase; DMSe: dimethyl selenide

*C. hupingshanensis* is a novel selenium hyperaccumulator plant in the Wuling mountain area of China with a content of its leaves as highest as 1427 mg Se kg^−1^, which was firstly discovered from trench of selenium diggings in Yutangba of Enshi City where has the highest grade selenium ore by resource development scientist, meanwhile it was found by taxonomist from Hupingshan national nature reserve of Hunan province [14, 15]. It has been reported that the genome length of *C. hupingshanensis* is 443.46 Mb (2n = 32), including 52,725 genes with a contig N50 of 1.23 Mb and a scaffold N50 of 24.41 Mb [16]. The genome and metabolome analysis of *C. hupingshanensis* seedlings treated with high concentrations of selenite showed that the flavonoid, glutathione, and lignin biosynthetic pathways may play important roles in stress induced by selenium [16]. Two cDNA libraries were constructed from the transcriptome of *C. hupingshanensis* seedlings treated with high concentrations of selenite, including 48,989 unigenes, with 39,579 expressed in the roots and 33,510 expressed in the leaves [17]. The results of RNA sequencing (RNA-Seq) and quantitative real-time PCR (RT-qPCR) showed that degradation of malformed selenoproteins, storage function, oxidation, transamination and selenation play very important roles in selenium tolerance [17]. The mechanism of selenium tolerance and hyperaccumulation in *C. hupingshanensis* was analyzed by multiple omics (Fig. 1). ATP sulfurylase (ATPS) is the first key enzyme to initiate the inorganic selenium assimilation pathway that has been identified in the genome, and the family member *ChATPS1-2* plays critical roles in stress induced by selenium [18].

The selenomethionine cycle (SeMTC) is the most important part of the metabolic pathway of selenium in plants [19]. Enzymes from multiple families participate in the cycle, including S-adenosyl-methionine synthase (MAT), SAM-dependent methyltransferase (MTase), S-adenosyl-homocysteine hydrolase (SAHH) and methionine synthase (MTR) [20, 21]. The enzyme from the MAT family-initiated cycle of selenomethionine converts SeMet to Se-adenosyl-L-selenomethionine (SeAM) with ATP [22]. The member from the superfamily of MTases catalyzes the second reaction to transfer the methyl groups of SeAM to the other pathways and form Se-adenosyl-L-selenohomocysteine (SeAH) in plants [23]. Then, SeAH is hydrolyzed to SeHcys and adenosine by enzymes from the SAHH family [24]. Finally, the formation of SeMet finished the cycle by the enzyme from the MTR family.

MAT is the only enzyme that converts SeMet to SeAM with ATP, which contains the SeMet binding site in the N-terminal domain and the ATP binding site in the C-terminal [25]. The genome of *Arabidopsis thaliana* contains 4 genes encoding MAT, *AtMAT1* and *AtMAT2*, which have similar sequences and are expressed in all organs [26]. *AtMAT3* is predominantly expressed in pollen and plays an essential role in the initial stage of pollen germination, and *AtMAT4* is enriched in all organs [27]. In addition, MAT is involved in the regulation of various stress responses. Existing research shows that MAT can enhance the tolerance of salt and drought stresses in *Tibetan wild barley* [28]. The overexpression of MAT in the calluses of tomatoes significantly enhanced tolerance to alkali stress by PA and H_2_O_2_ [29]. MTases are divided into three major families based on the chemical nature of the substrate: O-, N-, and C-methyltransferases [23]. O-methyltransferases (OMTs) act on the hydroxyl and carboxyl groups of phenylpropanoids, flavonoids, alkaloids and aliphatic substrates, and share domains for S-adenosyl-L-methionine (SAM) at the same time [30]. In plants, the major consumption of methyl from SAM is lignin biosynthesis. Therefore, caffeic acid O-methyltransferase (COMT) is an important MTase in plants [31–33]. The *A. thaliana* genome contains 17 *AtCOMT* genes that have a C-terminal catalytic domain of Methyltrans-2, including the conserved SAM/SAH binding domain and various substrate binding domains [34]. The *cost* knockout mutant exhibits less production of melatonin than the wild type in *A. thaliana*, which suggests that COMT also catalyzes the generation of melatonin [35]. Furthermore, COMT is involved in plant responses to stress by regulating the synthesis of lignin, such as in *A. thaliana* roots adapting to salt stress, *C. hupingshanensis* seedling roots adapting to selenium stress, and *Zea mays* leaves adapting to drought stress [17, 36–38]. SAHH is a key enzyme that maintains the potential of cell methylation and is the only enzyme to hydrolyze SeAH, which is the byproduct of the transfer of methyl groups [39]. Inhibition of this enzyme leads to an increase in S-adenosyl-L-homocysteine (SAH) accumulation, which inhibits the methylation pathway by a feedback inhibition mechanism [40–42]. The *A. thaliana* genome encodes two SAHH isoforms, of which *AtSAHH1* is essential for different developmental stages, and loss of *AtSAHH1* function results in developmental abnormalities in *A. thaliana*, including slow growth, root-hair development defects and low fertility [43, 44]. Furthermore, SAHH performs a crucial function in the plant response to pathogen infection, and SAHH can increase resistance to viral infection in transgenic tobacco plants [45]. MTR is the final enzyme of methionine (Met) synthesis in all living organisms [46]. Three isoforms of MTR were found in *A.thaliana*; *AtMTR1* and *AtMTR2* are present in chloroplasts for de novo Met synthesis, and *AtMTR3* is involved in the regeneration of Met from homocysteine produced during the activated methyl cycle in the cytosol [47]. One of the characteristics of AtMTR is a cationic loop (residues 507–529) in the N-terminal domain that combines with the first glutamyl residue of 5-methyltetrahydrofolate [48]. MTR is involved in not only methionine synthesis but also plant seed germination and various abiotic stresses [49]. For example, MTR promoted seed germination in *A. thaliana* by activating the GLR3.5 Ca^2+^ channel [50]. The levels of MTR were significantly increased in barley leaves under salt stress [51].

In the present study, SeMTC enzymes were comprehensively identified and analyzed for the first time in *C. hupingshanensis*, including the families of *ChMAT* (*C. hupingshanensis MAT*), *ChCOMT* (*C. hupingshanensis COMT*), *ChSAHH* (*C. hupingshanensis SAHH*) and *ChMTR* (*C. hupingshanensis MTR*). Phylogenetic relationships, conserved motifs, gene structure, chromosome location and protein characteristics were analyzed based on the genome of *C. hupingshanensis* to clarify the physicochemical properties and basic functions. In addition, molecular docking was used for the simulation of affinity to the selenium substrates. Finally, qRT-PCR was conducted to screen the main genes that responded to selenite stress, providing a molecular theoretical basis for the plant selenium metabolism.

## Methods

### Genome-wide identification of SeMTC genes

The gene annotation GTF file, nucleotide sequence FASTA file and protein sequence FASTA file of *C. hupingshanensis* were downloaded from the Genome Warehouse BIG Data Center (number PRJCA005533). The protein sequences of SeMTC in *A. thaliana* were obtained from The *Arabidopsis* Information Resource (TAIR, https://www.arabidopsis.org/), which was used as a query sequence for extracting the homologous protein sequence of SeMTC in *C. hupingshanensis* by the Blast Zone (BlastType: blastp, Outfmt: Table ) of TBtools software [52]. The obtained protein sequences of SeMTC in *C. hupingshanensis* were further verified using NCBI BLAST (https://blast.ncbi.nlm.nih.gov/blast/Blast.cgi). The conserved domains of SeMTC proteins in *C. hupingshanensis* were analyzed further using CD-search (https://www.ncbi.nlm.nih.gov/Structure/cdd/wrpsb.cgi). The physical and chemical properties of SeMTC proteins in *C. hupingshanensis*, including molecular weight (MW), isoelectric point (pI), grand average of hydropathicity (GRAVY), and instability index, were predicted and analyzed using the online tool ExPASy (https://web.expasy.org/protparam/) [53]. The subcellular localization of SeMTC in *C. hupingshanensis* was predicted by WoLF PSORT (https://wolfpsort.hgc.jp/).

### Chromosomal distribution and phylogenetic analysis of SeMTC genes

The chromosomal location information of ChSeMTC was obtained from the gene annotation GTF file of *C. hupingshanensis* for visualization by “Gene Location Visualize from GTF/GFF” of TBtools software [52]. The protein sequences of SeMTC in *Brassica napus*, *Brassica oleracea*, *Brassica rapa*, *Camelina sativa*, *Glycine max*, *Musa nana*, *Oryza sativa*, *Triticum aestivum*, and *Zea mays* were downloaded from NCBI (https://www.ncbi.nlm.nih.gov/) for multiple sequence alignment by Clustal W. A maximum likelihood (ML) tree with *C. hupingshanensis* and *A.thaliana* was constructed with all of the protein sequences using MEGA 11 [54], bootstrap = 1000 repetitions.

### Structure and functional characteristics analysis of SeMTC genes

The protein sequences of SeMTC in *C. hupingshanensis* and *A. thaliana* were submitted to the MEME website (http://meme-suite.org/tools/meme) to perform a conserved motif scan with the MEME motif set to 20. The conserved domain information of SeMTC in *C. hupingshanensis* and *A. thaliana* was obtained in the CD-search of NCBI’s conserved domain database (https://www.ncbi.nlm.nih.gov/Structure/bwrpsb/bwrpsb.cgi) by submitting the protein sequences. The intron-exon gene structure information of SeMTC genes was extracted from the GFF files of the *C. hupingshanensis* and *A. thaliana* genomes for further visualization by “Gene Structure View (advanced)” of TBtools [52]. The protein sequences of SeMTC in *C. hupingshanensis* and *A. thaliana* were aligned by ClustalW (https://www.genome.jp/tools-bin/clustalw). The result was further processed by ESPript 3.0 (https://espript.ibcp.fr/ESPript/cgi-bin/ESPript.cgi) to output the image [55].

### Homology modeling and ligand preparation

The best crystal structure was selected as the template for further validation in the SWISS-MODEL (https://swissmodel.expasy.org/) template library. The compounds Met, SAM, SAH and Hcys were selected from the ChemSpider database. The 3D structures of SeMet, SeAM, SeAH and SeHcys were downloaded in ChemSpider and then redrew it using ChemSketch. The protein active sites of SeMTC in *C. hupingshanensis* were predicted by PrankWeb [56].

### Molecular docking

Experiments involving the docking of SeMTC proteins in *C. hupingshanensis* with ligands were performed using AutoDock v4.2 [57]. The SeMTC proteins and ligand compounds were modified by AutoDock v4.2 including adding all hydrogens, incorporating nonpolar hydrogens and calculating Gasteiger charges. Subsequently, the molecular docking of SeMTC proteins and ligands was carried out using AutoDock v4.2 with the exhaustiveness setting at 10. The best aptamer conformations were selected based on the minimal binding energies. The ligand-protein interactions (hydrogen bonds and hydrophobic) were analyzed and visualized by PLIP and PyMol [58, 59]. The docking binding energy was visually analyzed by GraphPad Prism [60].

### Plant material and sample preparation

The seeds of *C. hupingshanensis* were collected from the 5th floor of the Key Laboratory of Hubei University for Nationalities, Enshi, Hubei Province in June 10, 2022. The *C. hupingshanensis* seeds were planted in a room where the temperature was 22 ± 1 °C, the light period was 16 h and the irradiance was 1500 mol^−2^ ms^−1^ in June 25, 2022. Forty-five seedlings approximately 10 cm tall and 4 months old were selected as samples, and the roots were washed with melanchorite and balanced in Hoagland’s solution for two days. The samples were treated with different concentrations of selenium (100 µg Se L^−1^ and 80,000 µg Se L^−1^), and 0 µg Se L^−1^ was the control group. The sodium selenite (Na_2_SeO_3_) as the selenium source. The leaves on the third node from the top and roots of 9 seedlings were separated at 0, 3, 6 and 24 h. All samples were harvested, snap-frozen using liquid nitrogen and kept at -80 °C until RNA extraction. Three biological replicates of each sample were collected for analysis.

### Gene expression analysis

The total RNA of roots and leaves was extracted by the TransZolTM Up Plus RNA Kit. The RNA concentration and quality were detected by a NanoDrop 2000. 1% agarose gel electrophoresis was used to detect RNA integrity and genomic DNA contamination. Residual genomic DNA in RNA samples was removed by RNase-free DNase. Real-time PCR was carried out on ABI StepOne Plus. The expression of target genes in the samples was detected using the Hieff qPCR SYBR Green Mix commercial kit, and gene expression was calculated using the 2^−ΔΔCT^ method [61]. The results were analyzed and graphical representation was carried out using GraphPad Prism, and the significance was analyzed by the LSD test of single-factor ANOVA (*p* < 0.05) [60]. All analyses were performed in triplicate. The primers used for the qRT-PCR analysis are listed in Table S2.

## Results

### Identification and analysis of SeMTC genes in *C. Hupingshanensis*

A total of 45 genes were identified in *C. hupingshanensis* (the Genome Warehouse BIG Data Center accession number PRJCA005533) by comparison with the genome sequences of *A. thaliana*, including 7 *ChMAT* genes, 27 *ChCOMT* genes, 4 *ChSAHH* genes and 7 *ChMTR* gene. The characteristics of each gene, such as molecular weight, number of amino acids, grand average of hydropathicity, subcellular localization, and isoelectric points, are listed in Table 1. The gene coding sequence and protein sequence can be found in S1.

The ChMAT protein sequences exhibited a range in length, spanning from 390 to 393 amino acids. Additionally, their molecular weights varied between 43.1 and 43.9 kDa. These proteins were primarily found in the cytosol and cytoskeleton. The length of the ChCOMT protein sequences ranged from 230 to 381 amino acids, and the molecular weights ranged from 25.5 to 42.4 kDa, mainly located in the cytosol, chloroplast, Golgi apparatus and extracellular. ChSAHH has 485 amino acids and molecular weights from 53.3 to 53.4 kDa, mainly located in the cytosol. The ChMTR protein sequences exhibited variations in their lengths, spanning from 765 to 812 amino acids. Additionally, their molecular weights ranged between 84.3 and 90.5 kDa. These proteins were primarily localized in the cytosol, chloroplast, and mitochondrion. The isoelectric points of most genes involved in the SeMTC are less than 7, indicating that amino acids are generally acidic.

**Table 1:** The basic physicochemical properties of genes involved in the SeMTC.

	Gene ID	Gene name	Length (aa)	pI	MW (Da)	Instability index	Subcellular localization	GRAVY
MAT	Chu000144	ChMAT1-1	393	5.51	43229.07	22.47	Cytosol	-0.328
	Chu016972	ChMAT1-2	393	5.59	43135.06	21.54	Cytoskeleton	-0.305
	Chu010190	ChMAT2-1	393	5.67	43226.07	25.89	Cytoskeleton	-0.334
	Chu039088	ChMAT2-2	393	5.58	43212.00	24.40	Cytoskeleton	-0.333
	Chu008039	ChMAT3-1	390	5.87	42554.57	26.01	Cytoskeleton	-0.243
	Chu034452	ChMAT3-2	390	5.87	42554.57	26.01	Cytoskeleton	-0.243
	Chu049411	ChMAT4	393	5.52	42920.71	27.34	Cytoskeleton	-0.295
COMT	Chu006643	ChCOMT1-1	358	5.82	39193.43	33.15	Cytosol	0.047
	Chu011396	ChCOMT1-2	364	4.98	39733.94	34.55	Cytosol	0.055
	Chu011397	ChCOMT1-3	328	6.25	36427.22	45.36	Cytosol	0.002
	Chu011575	ChCOMT1-4	363	5.58	39497.78	33.16	Cytosol	0.064
	Chu018237	ChCOMT1-5	367	5.6	40279.67	33.03	Cytosol	0.072
	Chu027749	ChCOMT1-6	381	5.62	42019.61	35.22	Cytosol	-0.011
	Chu040299	ChCOMT1-7	346	5.75	37635.85	33.91	Cytosol	0.133
	Chu040466	ChCOMT1-8	364	5.49	39775.12	38.39	Cytosol	0.02
	Chu017536	ChCOMT2	230	4.83	25516.61	34.39	Cytosol	0.164
	Chu000540	ChCOMT3	365	5.72	40676.02	32.49	Extracellular	-0.17
	Chu006752	ChCOMT4	262	6.07	28911.64	32.66	Golgi apparatus	0.139
	Chu004432	ChCOMT5-1	353	5.34	39455.98	30.08	Cytosol	0.214
	Chu032308	ChCOMT5-2	381	5.24	42461.45	38.59	Cytosol	0.107
	Chu004496	ChCOMT7	370	4.99	40641.84	23.10	Cytosol	0.071
	Chu025446	ChCOMT8	359	4.96	39687.28	44.36	Cytosol	-0.153
	Chu001811	ChCOMT9-1	376	5.05	40970.14	26.79	Cytosol	0.07
	Chu015268	ChCOMT9-2	376	5.62	41147.51	26.25	Cytosol	0.101
	Chu015269	ChCOMT9-3	376	5.11	40976.13	27.26	Cytosol	0.068
	Chu015270	ChCOMT9-4	373	5.2	40773.98	26.60	Cytosol	0.075
	Chu002772	ChCOMT12-1	262	6.23	28757.00	33.34	Cytosol	0.072
	Chu014285	ChCOMT12-2	354	5.43	39055.90	36.58	Cytosol	0.082
	Chu001812	ChCOMT13	342	5.37	37320.13	28.60	Cytosol	0.123
	Chu026268	ChCOMT16-1	278	9.08	31018.98	38.18	Chloroplast	-0.043
	Chu047315	ChCOMT16-2	255	9.28	29327.52	44.06	Chloroplast	0.084
	Chu017535	ChCOMT17-1	368	5.24	40781.98	37.38	Cytosol	-0.02
	Chu027003	ChCOMT17-2	368	5.3	40916.39	43.02	Cytosol	-0.005
	Chu027004	ChCOMT17-3	372	5.15	41212.34	39.65	Cytosol	-0.098
SAHH	Chu019546	ChSAHH1-1	485	5.66	53410.58	32.61	Cytosol	-0.136
	Chu029073	ChSAHH1-2	485	5.61	53387.50	32.45	Cytosol	-0.139
	Chu021796	ChSAHH2-1	485	5.58	53402.41	35.06	Cytosol	-0.131
	Chu048579	ChSAHH2-2	485	5.57	53346.35	34.97	Cytosol	-0.12
MTR	Chu036986	ChMTR1-1	765	5.98	84598.85	37.49	Cytosol	-0.154
	Chu043295	ChMTR1-2	765	5.97	84307.45	37.20	Cytosol	-0.152
	Chu043317	ChMTR1-3	765	5.97	84321.48	36.91	Cytosol	-0.152
	Chu023711	ChMTR2-1	765	5.8	84688.86	35.61	Chloroplast	-0.17
	Chu047804	ChMTR2-2	765	5.94	84771.96	36.76	Cytosol	-0.181
	Chu037265	ChMTR3-1	812	8.41	90553.32	39.05	Mitochondrion	-0.149
	Chu043731	ChMTR3-2	812	8.47	90376.07	40.51	Mitochondrion	-0.14

### Chromosomal distribution of SeMTC genes in *C. Hupingshanensis*

**Fig. 2:**
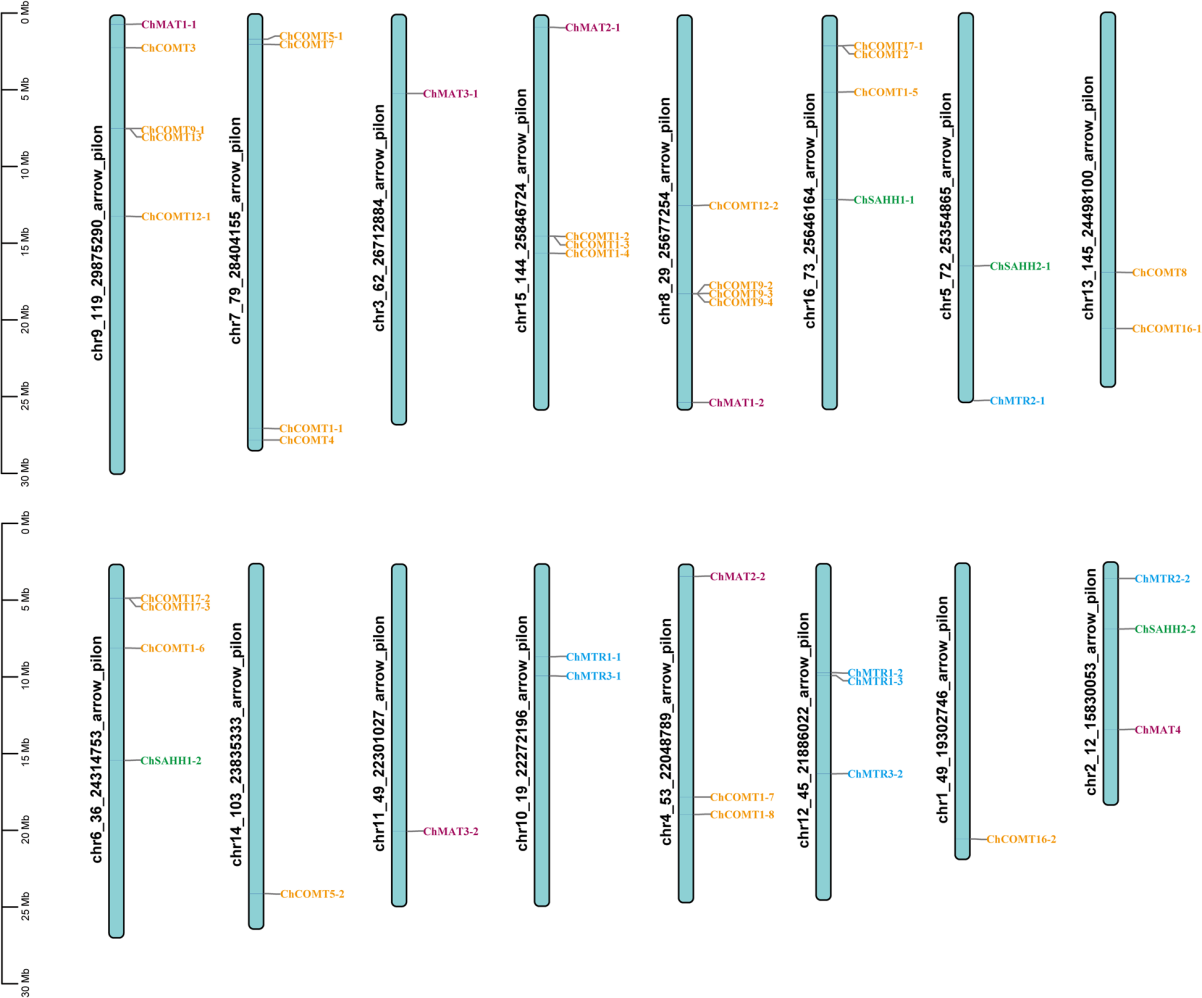
Chromosomal distribution of SeMTC genes in *C. hupingshanensis*. The chromosome numbers are shown on the left side of each strip

The SeMTC genes are randomly distributed on chromosomes 1–16 of *C. hupingshanensis* (Fig. 2). Chromosomes 8 and 9 carried the highest number of 5 genes belonging to the *ChCOMT* and *ChMAT* families. Chromosomes 3 and 11 had a single gene of the *ChMAT3* family. Chromosomes 1 and 14 also had a single gene of the *ChCOMT* family. The members of the *ChCOMT* family were widely distributed on 10 different chromosomes except on chromosomes 2, 3, 5, 10, 11 and 12. The close association of *ChCOMT* was observed in chromosome numbers 1, 7, 13 and 14. A close association of *ChMTR* was observed in chromosome numbers 10 and 12. Gene duplication has been recognized as one of the major factors for gene family expansion. A duplicated gene can be retained as is and perform the same function as an identical copy or it can evolve into a gene with a novel function. A close linkage was found in most genes of *ChCOMT*, indicating that members of the *ChCOMT* gene family have experienced tandem repeats during evolution. This observation sheds light on the evolutionary history and potential functional implications of SeMTC gene families in *C. hupingshanensis*.

### Phylogenetic analysis of SeMTC genes in C. Hupingshanensis

**Fig. 3:**
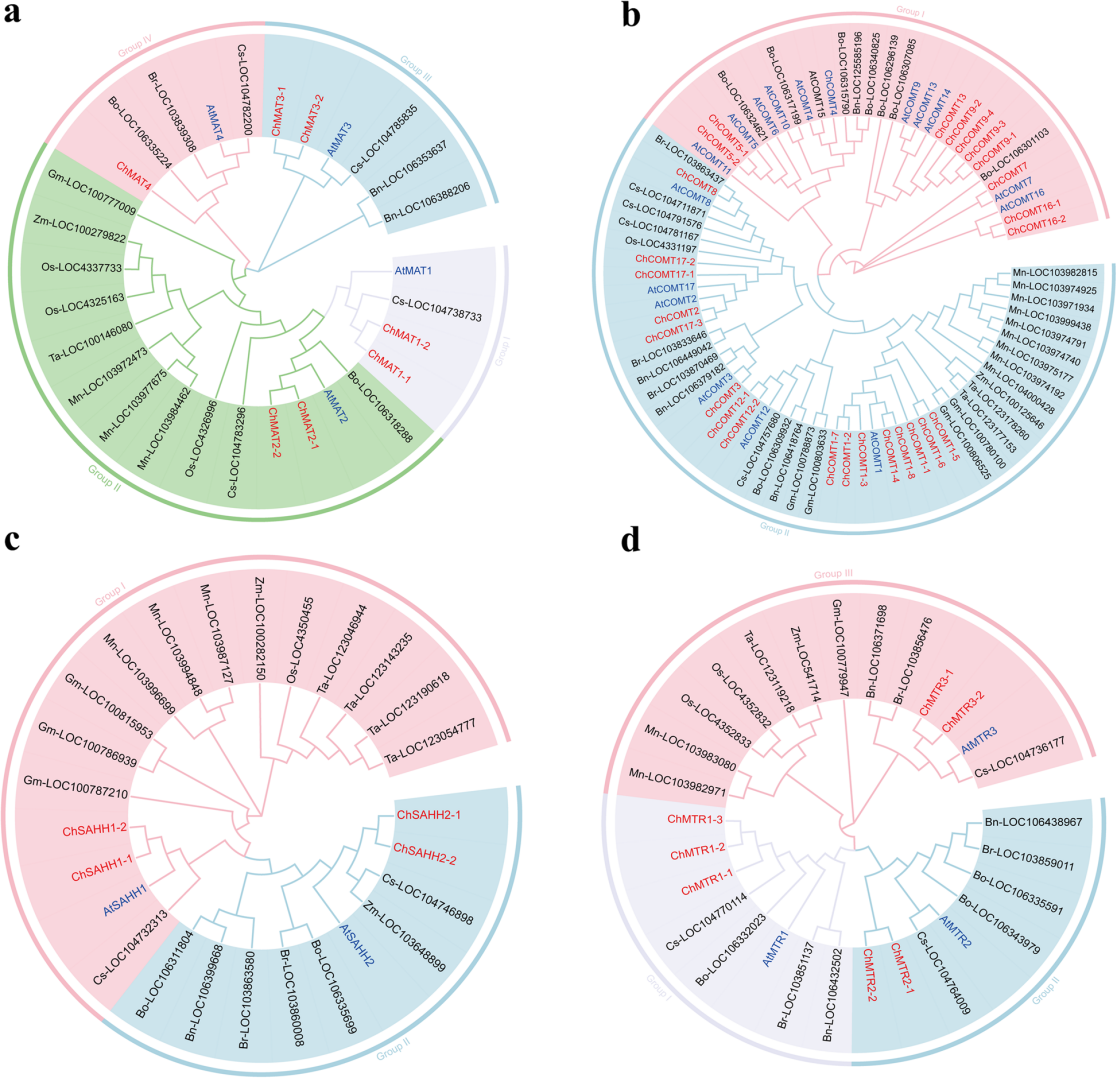
Phylogenetic tree of SeMTC genes. The phylogenetic tree from *Brassica napus* (Bn), *Brassica oleracea* (Bo), *Brassica rapa* (Br), *Camelina sativa* (Cs), *Glycine max* (Gm), *Musa nana* (Mn), *Oryza sativa* (Os), *Triticum aestivum* (Ta), *Zea mays* (Zm), *Arabidopsis thaliana* (At) and *C. hupingshanensis* (Ch). **a** The phylogenetic tree of MTR. **b** The phylogenetic tree of MAT. **c** The phylogenetic tree of COMT. **d** The phylogenetic tree of SAHH.

To better understand the phylogenetic relationship of SeMTC genes, phylogenetic trees of these genes in *C. hupingshanensis* and other plants, including dicotyledons (*A.thaliana*, *Brassica napus*, *Brassica oleracea*, *Brassica rapa*, *Camelina sativa*) and monocots (*Glycine max*, *Musa nana*, *Oryza sativa*, *Triticum aestivum*, *Zea mays*) were constructed by maximum likelihood (ML) according to the bootstrap value and phylogenetic topology (Fig. 3). The *ChMAT* gene family was clustered into 4 subgroups, with group IV being the smallest subset consisting of a single member (Fig. 3a). On the other hand, the *ChCOMT* gene family was clustered into 2 subgroups, with *ChCOMT1* having 8 members (Fig. 3b). Notably, the *ChCOMT16* and *ChCOMT7* subsets were distantly related to the other subsets, forming a relatively independent clade. This suggests the possibility of functional differentiation among these subsets. In contrast, the *ChSAHH* gene family had the fewest members, with only four members divided into 2 subgroups (Fig. 3c). As for the *ChMTR* gene family, it was clustered into 4 subgroups based on bootstrap values and phylogenetic topology (Fig. 3d). Group I was the largest subset with 3 members, while the other two groups had 2 members. The phylogenetic tree revealed a close relationship between the SeMTC genes in *C. hupingshanensis* and those in *A. thaliana*. This finding suggests a potential evolutionary connection between the two species in terms of the SeMTC gene family.

### Structure and functional characteristics analysis of SeMTC genes

**Fig. 4:**
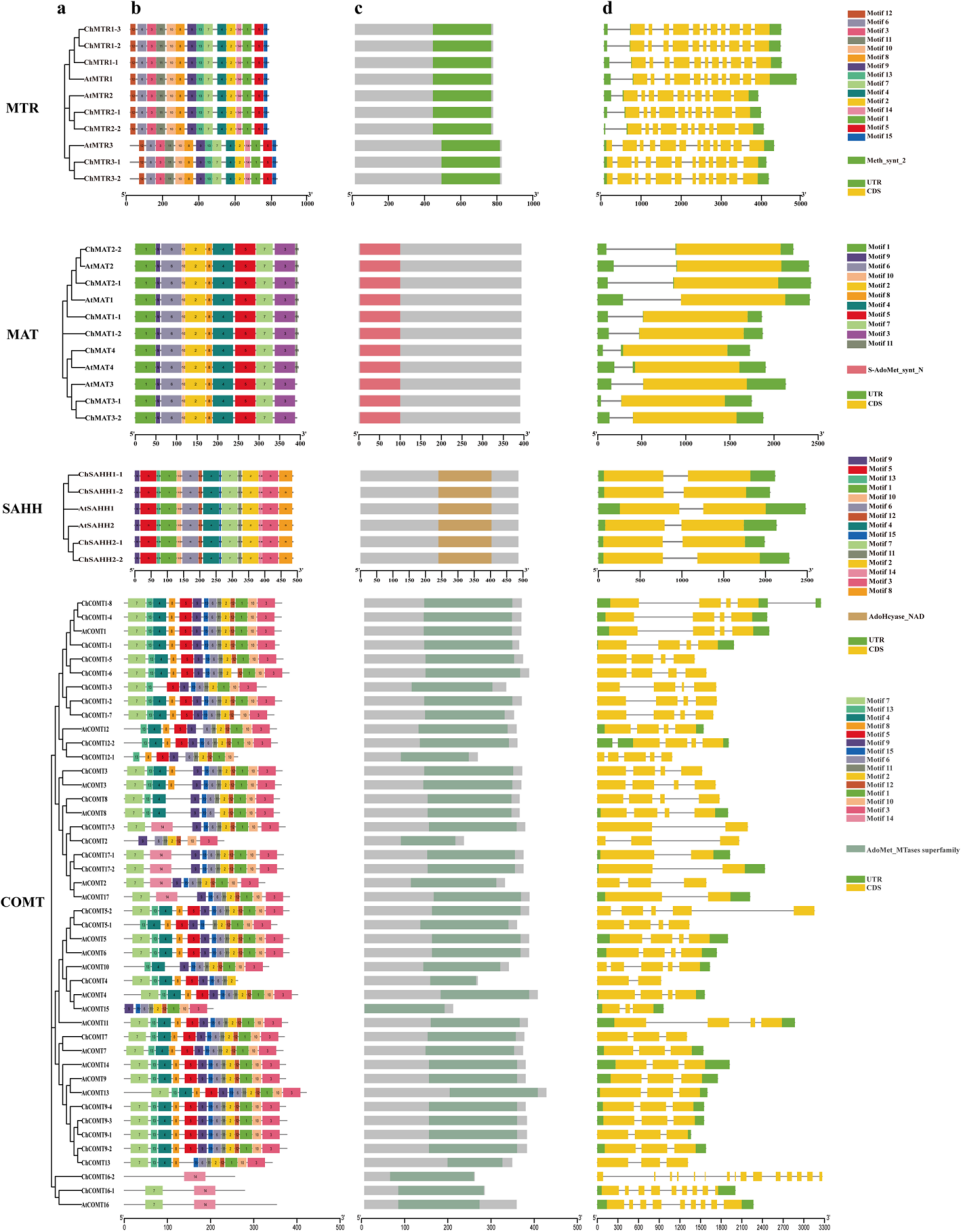
Phylogenetic trees, motif, domain, and gene structure of the SeMTC genes. **a** The phylogenetic tree; **b**,**c** Conserved motifs and domains of the proteins, different colors represent different motifs or domains. **d** Exon-intron structures; exons are indicated by yellow boxes, and introns are indicated by lines

A simplified maximum likelihood phylogenetic tree was constructed using the protein sequences of SeMTC genes from *C. hupingshanensis* and *A. thaliana* to identify protein motifs, conserved domains, and gene structures (Fig. 4). In the ChMAT family, 11 motifs were predicted. ChMAT1 and ChMAT2 showed all 11 motifs in the same order, while ChMAT3 lacked motif 11, suggesting unique evolutionary functions. The S-adenosylmethionine synthase N-terminal domain structure (S-AdoMet_synt_N, PF00438) was present in all ChMAT proteins, which is Met-binding motif domain (^119^GAGDQG^124^) and ATP-binding motif domain (^266^GGGAFSGKD^275^) (Fig. S1) [62]. Additionally, all *ChMAT* genes contain coding regions (CDS) and untranslated regions (UTR), with *ChMAT2* having a longer intron sequence.

Most members of the ChCOMT family shared similar conserved motifs, in which motif 2 was present in all ChCOMT proteins, forming part of the conserved structural domain, which is LVDVGG (Fig. S2). The SAM-dependent methyltransferase transfer domain structure (AdoMet_MTases superfamily, PF00891), which exhibits five conserved motifs: LVDVGGGxG, GINFDLPHV, EHVGGDMF, NGKVI, and GGKERT, existed in all ChCOMT proteins [63]. In the ChCOMT16 subgroup, the conserved motifs show that the Asp^96/196^ residue in motif 7 and motif 14 is replaced by Arg (Fig. S2). During the course of evolution, the genes encoding ChCOMT have undergone significant divergence, particularly in the CDS and UTR.

All four members of the ChSAHH gene family exhibited a high level of conservation, containing all 15 conserved motifs in the same order. Each gene contained one intron and two exons. The conserved domain was AdoHcyase_NAD structures (PF00670), including ^62^MTIQTAVLIETLTALGAEVRWCSC^85^ and ^251^GLMRATDVMIAG KVAVI^272^ (Fig. S3). The Ile^272^ residue in the second binding domain in ChSAHH1-2 was replaced by Val.

The ChMTR family exhibited a total of 15 identified motifs, and all proteins displayed a conserved methionine synthase domain structure (Meth_synt_2, PF01717). Additionally, an important motif (^507^FAFTANGWVQSYGSRCVKPPVIY^529^, a cationic loop) was present in this domain and served as a binding site for 5-methyltetrahydrofolate substrate [64, 65]. Interestingly, specific residue substitutions were observed in ChMTR3-1, ChMTR3-2, ChMTR2-1, and ChMTR2-2 (Fig. S4). In terms of gene structures, the ChMTR genes exhibited a high degree of similarity, particularly with regard to the coding regions, which consistently demonstrated uniform length and structure.

### Tertiary structures prediction of SeMTC enzymes

**Fig. 5:**
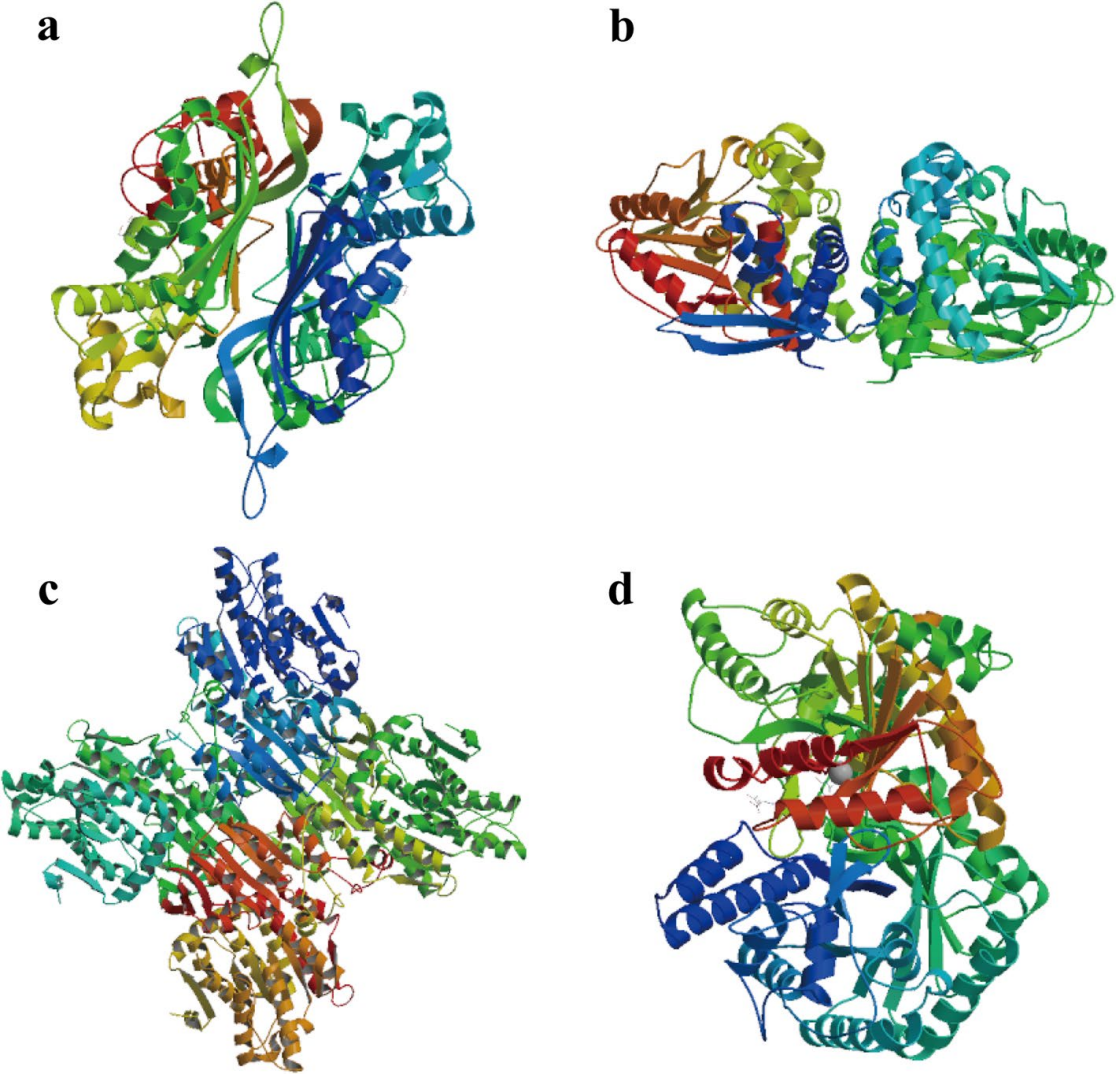
Predicted 3D structures of proteins by the SWISS-MODEL server. **a** ChMAT1-1. **b** ChCOMT1-1. **c** ChSAHH1-1. **d** ChMTR1-1.

PDB and SWISS-MODEL library BLAST searches were performed to identify the appropriate templates for the SeMTC enzymes. Proteins with the highest similarity scores (ranging from 35.00 to 97.96%) were selected as templates (Table 2). The crystal structure of S-adenosylmethionine synthase 1 (SMTL ID: 6vcx.1. A) of *A. thaliana* was used as the template for ChMAT (Fig. 5, Fig. S5). The O-methyltransferase in complex with S-adenosylhomocysteine (SMTL ID: 6i71.1. A) of *Fragaria ananassa* was used as the template for ChCOMT (Fig. 5, Fig. S6). There was inferior quality in that the ChCOMT16-1, ChCOMT16-2, ChCOMT17-1, ChCOMT17-2 and ChCOMT17-3 templates in 6i71.1.A. Therefore, the O-methyltransferase (SMTL ID: 3cbg.1. A) of *Cyanobacterium* was used as the template for ChCOMT16-1 and ChCOMT16-2, and the crystal structure of O-methyltransferase (SMTL ID: 5icc.1. A) of (S)-norcoclaurine was used as the template for ChCOMT17-1, ChCOMT17-2 and ChCOMT17-3. The crystal structure of S-adenosyl-L-homocysteine hydrolase (SMTL ID: 3ond.1. A) of *Lupinus luteus* was used as the template for ChSAHH (Fig. 5, Fig. S7). The cobalamin-independent methionine synthase (SMTL ID: 1u1j.1.A) of *A. thaliana* was used as the template for ChMTR (Fig. 5, Fig. S8). The tertiary structure prediction of proteins had high QMEAN and GMQE scores, indicating that the predicted structures were likely of high quality. GMQE values are all between 0 and 1 and close to 1, indicating the high quality of modeling expectations. QMEAN is also close to the interval of -4-0 and close to 0, which proves that the modeling matching degree is very high. These results indicate that the models obtained with homology models are acceptable and can be used for further molecular docking.

**Table 2:** Validation of the modeled structures of methionine cycle enzyme proteins

Gene	Template	Sequence Identity	Coverage	GMQE	QMEAN
ChMAT1-1	6vcx.1.A	97.96%	1	0.98	0.85
ChMAT1-2	6vcx.1.A	97.46%	1	0.97	0.93
ChMAT2-1	6vcx.1.A	96.44%	1	0.97	1.07
ChMAT2-2	6vcx.1.A	96.69%	1	0.98	1.02
ChMAT3-1	6vcx.1.A	89.46%	1	0.96	0.83
ChMAT3-2	6vcx.1.A	89.46%	1	0.96	0.83
ChMAT4	6vcx.1.A	92.11%	1	0.97	1.17
ChCOMT1-1	6i71.1.A	70.85%	0.96	0.85	-1
ChCOMT1-2	6i71.1.A	69.05%	0.96	0.84	-0.16
ChCOMT1-3	6i71.1.A	55.93%	0.79	0.72	-1.98
ChCOMT1-4	6i71.1.A	80.80%	0.96	0.88	0.02
ChCOMT1-5	6i71.1.A	73.58%	0.96	0.86	0.2
ChCOMT1-6	6i71.1.A	74.43%	0.92	0.83	-0.14
ChCOMT1-7	6i71.1.A	68.69%	0.95	0.82	-0.53
ChCOMT1-8	6i71.1.A	81.95%	0.96	0.88	0.17
ChCOMT2	6i71.1.A	34.43%	0.92	0.66	-4.29
ChCOMT3	6i71.1.A	46.42%	0.96	0.78	-1.23
ChCOMT4	6i71.1.A	44.21%	0.89	0.72	-2.28
ChCOMT5-1	6i71.1.A	49.71%	0.97	0.80	-0.74
ChCOMT5-2	6i71.1.A	48.86%	0.92	0.76	-2.02
ChCOMT7	6i71.1.A	44.90%	0.93	0.74	-1.96
ChCOMT8	6i71.1.A	37.68%	0.96	0.75	-2.25
ChCOMT9-1	6i71.1.A	47.98%	0.92	0.75	-1.76
ChCOMT9-2	6i71.1.A	47.55%	0.92	0.75	-1.41
ChCOMT9-3	6i71.1.A	47.84%	0.92	0.75	-1.35
ChCOMT9-4	6i71.1.A	47.40%	0.93	0.76	-1.36
ChCOMT12-1	6i71.1.A	46.15%	0.94	0.78	-1.81
ChCOMT12-2	6i71.1.A	46.70%	0.99	0.81	-1.23
ChCOMT13	6i71.1.A	46.52%	0.92	0.73	-1.87
ChCOMT16-1	3cbg.1.A	46.05%	0.98	0.77	-0.98
ChCOMT16-2	3cbg.1.A	38.99%	0.85	0.68	-1.83
ChCOMT17-1	5icc.1.A	35.00%	0.92	0.69	-2.94
ChCOMT17-2	5icc.1.A	35.19%	0.93	0.70	-2.91
ChCOMT17-3	5icc.1.A	36.47%	0.91	0.68	-2.83
ChSAHH1-1	3ond.1.A	92.58%	1	0.97	0.39
ChSAHH1-2	3ond.1.A	92.16%	1	0.97	0.47
ChSAHH2-1	3ond.1.A	91.55%	1	0.97	0.30
ChSAHH2-2	3ond.1.A	91.34%	1	0.97	0.30
ChMTR1-1	1u1j.1.A	97.12%	1	0.91	-2.08
ChMTR1-2	1u1j.1.A	97.65%	1	0.91	-2.68
ChMTR1-3	1u1j.1.A	97.52%	1	0.91	-2.67
ChMTR2-1	1u1j.1.A	92.16%	1	0.9	-2.56
ChMTR2-2	1u1j.1.A	91.37%	1	0.9	-2.31
ChMTR3-1	1u1j.1.A	80.29%	0.94	0.83	-2.17
ChMTR3-2	1u1j.1.A	80.42%	0.94	0.83	-2.31

### Molecular docking

**Fig. 6:**
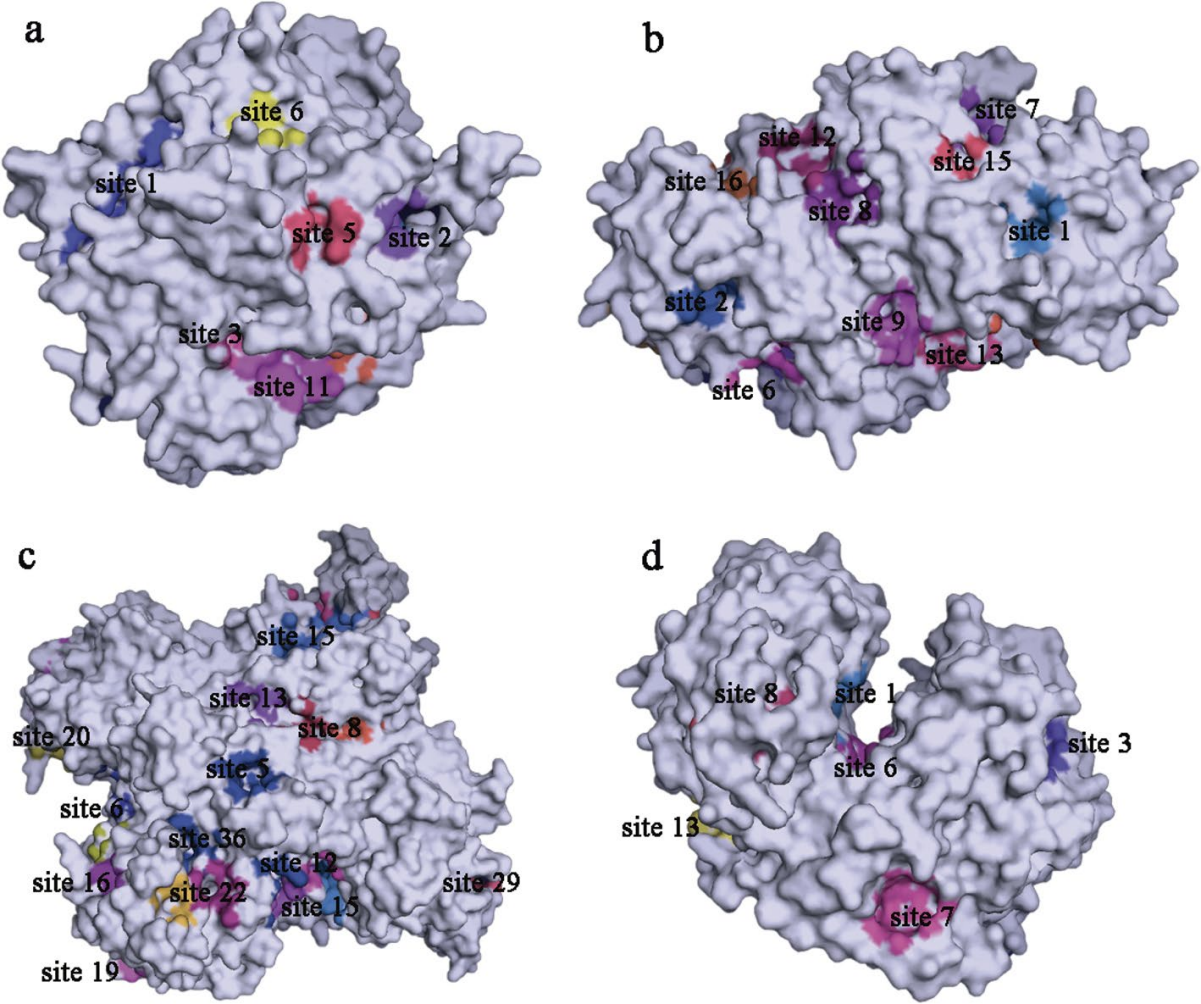
Visualization of some of the predicted ligand-binding sites for protein by PrankWeb. **a** ChMAT1-1. **b** ChCOMT1-1. **c** molChSAHH1-1. **d** ChMTR1-1.

**Fig. 7:**
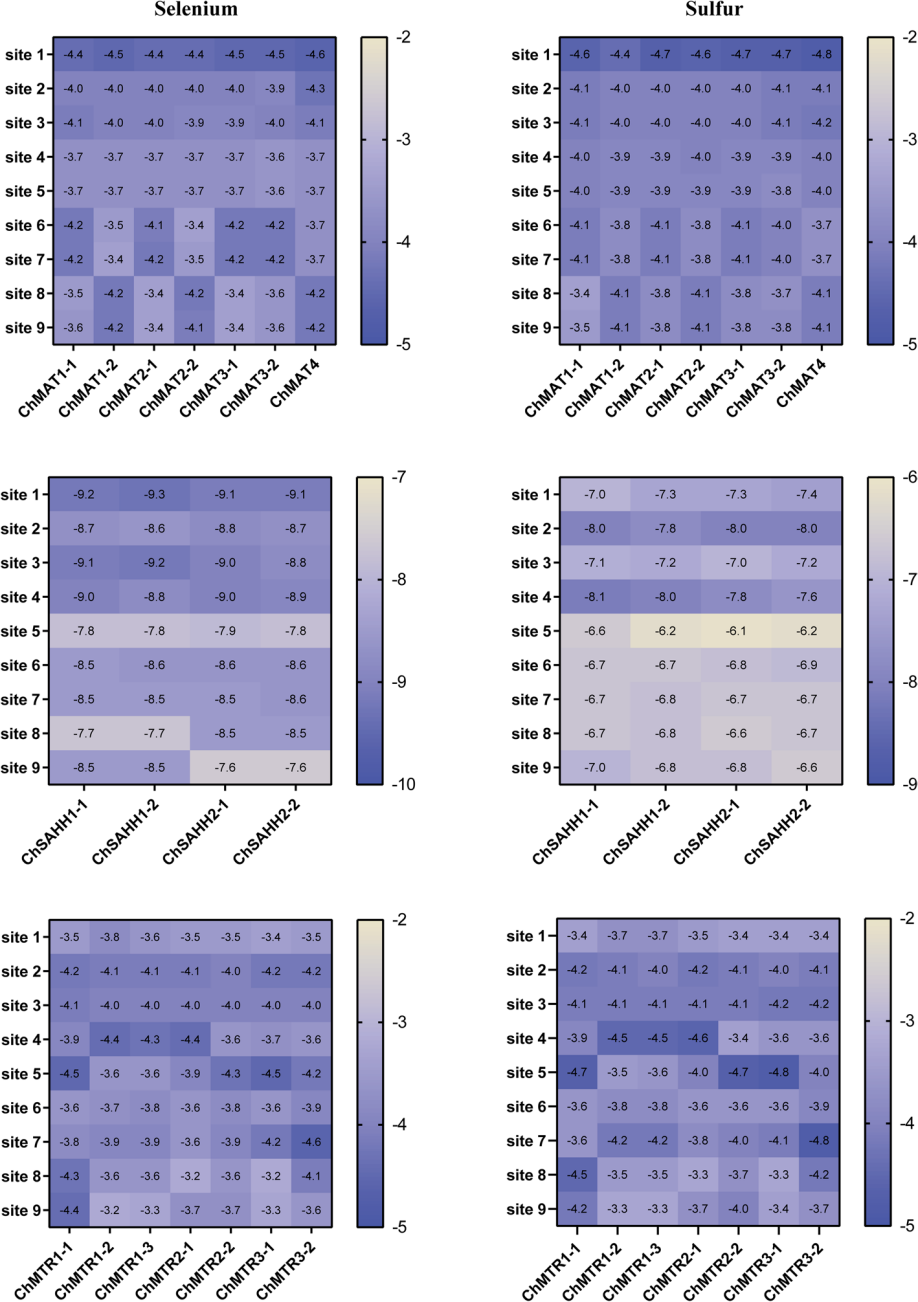
Binding energies of ChMAT and SeMet/Met, ChSAHH and SeAH/SAH, ChMTR and SeHcys/Hcys. The bottom of the heat map represents different genes, and the vertical coordinates represent the ligand binding sites. The value represents the binding energy shown by the ligand-protein docking, unit: kcal·mol^−1^

**Fig. 8:**
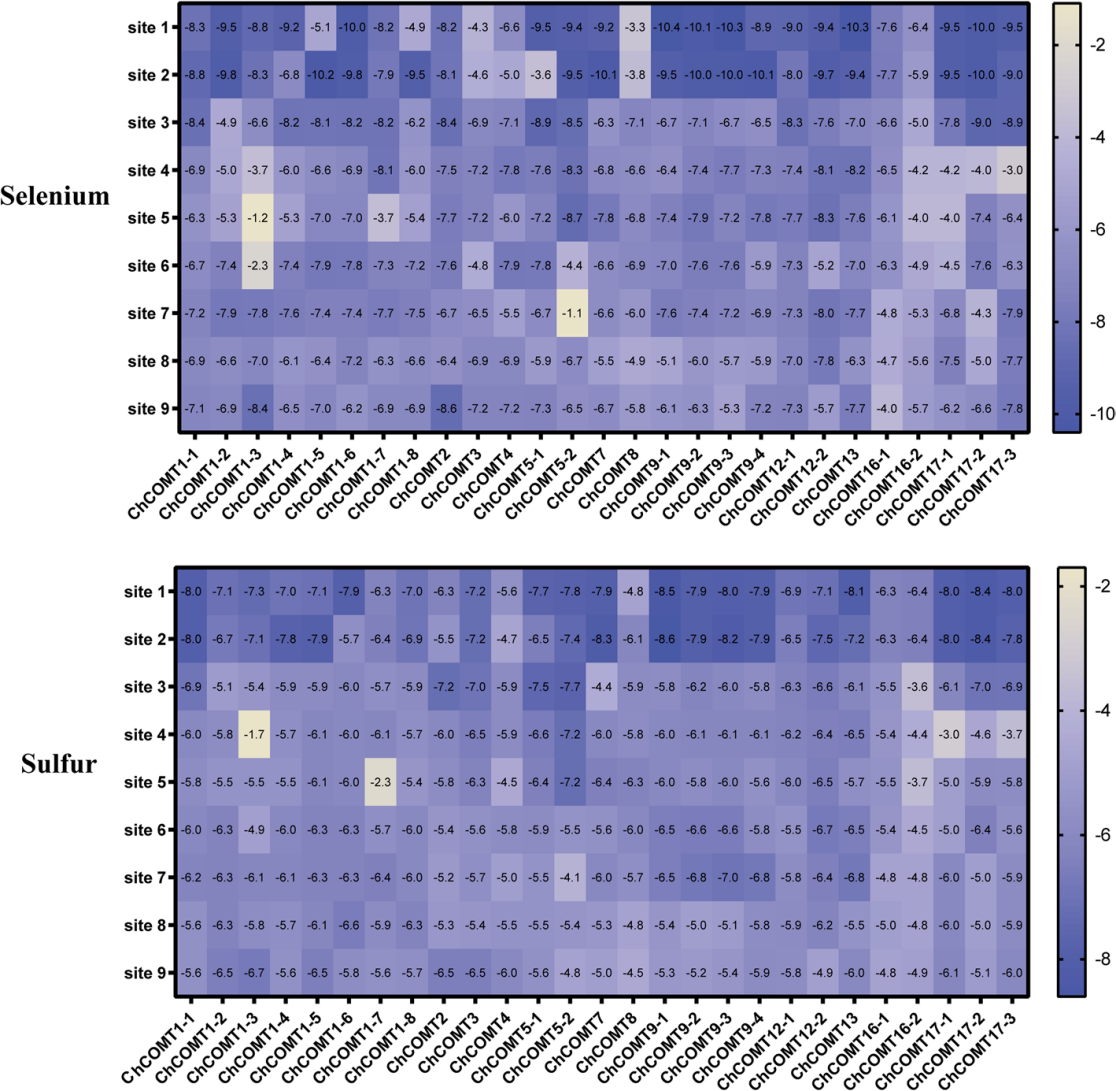
Binding energies of ChCOMT and SeAM/SAM. The bottom of the heat map represents different genes, and the vertical coordinates represent the ligand binding sites. The value represents the binding energy shown by the ligand-protein docking, unit: kcal·mol^−1^

The ligand-binding sites of SeMTC enzymes were predicted by the Prankweb online server. Several ligand-binding sites were predicted for each enzyme, ranging from 1 to 31(Fig. 6). The top 9 binding sites with higher scores were selected for molecular docking, and the results showed that the binding energies of ChMAT with SeMet/Met and ChMTR with SeHcys/Hcys ranged from − 2 to − 5 kcal·mol^−1^, and the binding energies of ChSAHH with SeAH/SAH ranged from − 6.1 to -9.3 kcal·mol^−1^ (Fig. 7). The affinity of ChMAT4 for SeMet (-4.6 kcal·mol^−1^) and Met (-4.8 kcal·mol^−1^) was stronger than that of the other ChMAT genes. The affinity of ChSAHH was significantly higher for SeAH (-7.6 to -9.3 kcal·mol^−1^) than for SAH (-6.1 to -8.1 kcal·mol^−1^). The affinity of ChMTR3-2 for SeHcys (-4.6 kcal·mol^−1^) and Hcys (-4.8 kcal·mol^−1^) was stronger than that of the other ChMTR genes. It is worth noting that the affinity of ChCOMT for SeAM (-1.1 to -10.4 kcal·mol^−1^) was significantly higher than that for SAM (-1.7 to -8.5 kcal·mol^−1^), and ChCOMT9-1 had the strongest affinity (Fig. 8).

**Fig. 9:**
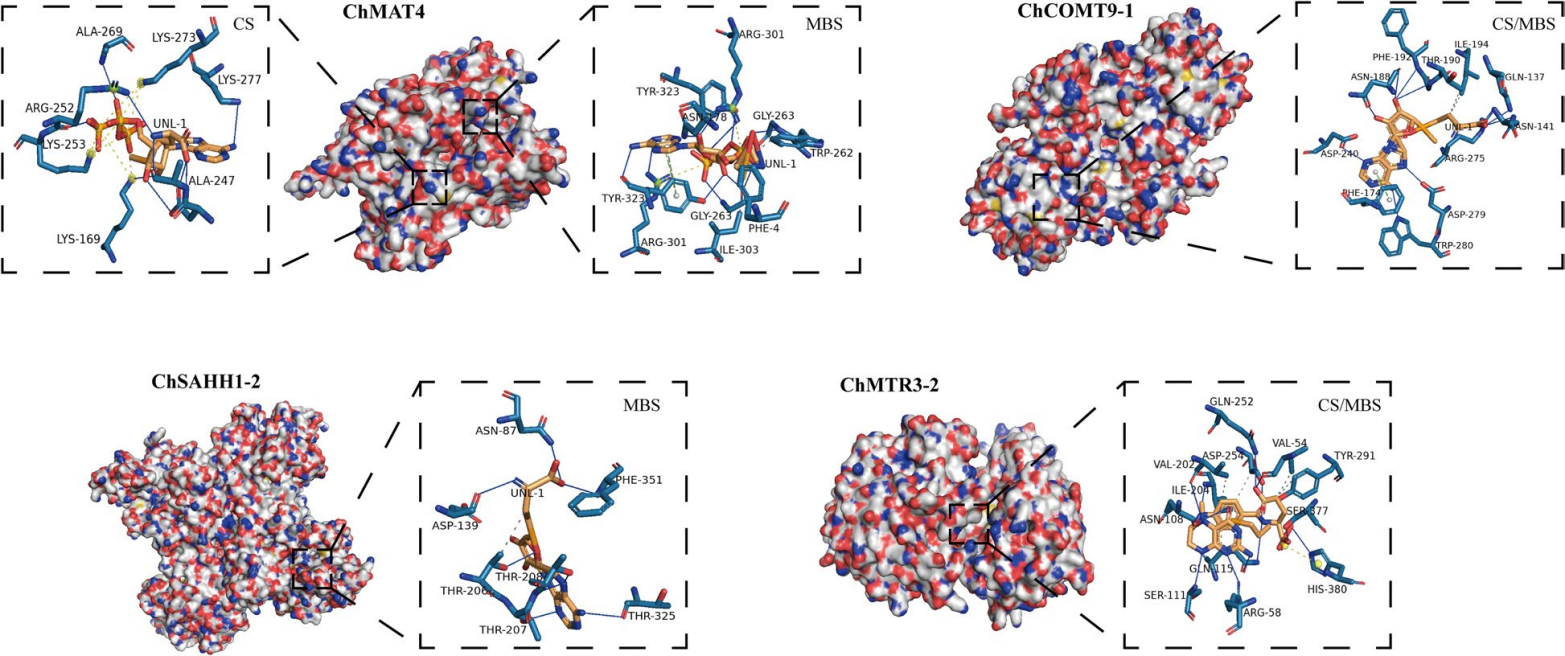
Interactions of the SeMTC enzymes and ligands. The left panel is the overall view, and the right panel is the focused view. The SeMTC enzymes are shown on the surface, the amino acid residues at the binding site are gray-blue, and the ligands are heavily yellow. The gray dotted line represents hydrophobic interactions, the solid blue line represents the hydrogen bond, the dashed yellow line represents the salt bridge, and the red dashed line represents a π-cation interaction. ChMAT4: Interactions of the binary ChMAT-ATP complex with SeMet. ChCOMT9-1: Interactions of the binary ChCOMT with SeAM. ChSAHH1-2: Interactions of the binary ChSAHH with SeAH. ChMTR3-2: Interactions of the binary ChMTR-5-methyltetrahydrofolate complex with SeHcys. CS: putative binding mode of SeMTC enzymes and ligands to model the protein structure at the catalytic site. MBS: SeMTC enzymes and ligands are in a putative binding mode that mimics the protein structure at the site of minimum binding energy, the site of maximum affinity binding

To provide insight into the interactions between protein and ligand, molecular docking was performed to determine the binding affinities between them and predict binding modes. Hydrogen-bond interactions were found to be necessary for the interactions of binary complexes ChMAT-ATP with SeMet (Fig. 9, Fig. S9). The catalytic site (CS) and the maximum affinity binding site (MBS) are similar. ChMAT is surrounded by Gly^263^ and Asp^178^ in the MBS. Ala^269^ and Lys^273^ are key active site residues in the CS. The amino acid residues (Leu^221/231^ and Gly^207/249^) involved in the interaction of ChCOMT with SeAM were found in the CS/MBS (Fig. 9, Fig. S10). Notably, the catalytic domain of ChSAHH was subdivided into numerous ligand-binding sites by PrankWeb, leading to the prediction of the SeAH interaction with ChSAHH in MBS through molecular docking, with key binding amino acids residues identified as Asn^87^, Asp^139^ and Thr^206/207/208/325^(Fig. 9, Fig. S11). The interaction of 5-methyltetrahydrofolate with SeHcys involves specific amino acid residues of ChMTR, namely Ile^204^, Ser^111/329/377^, Asp^70/206/254^, and His^329/332/380^ (Fig. 9, Fig. S12).

### Expressions analysis of SeMTC enzymes in different tissues under Se stress

**Fig. 10:**
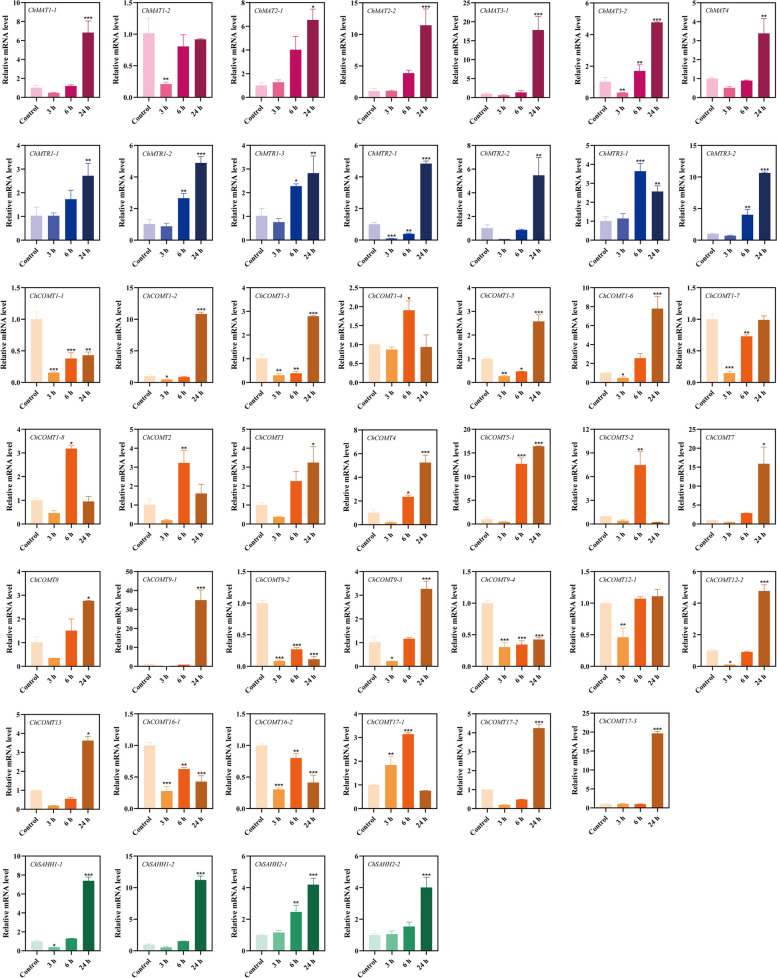
Expression of *ChSeMTC* genes in leaves under low-concentration selenium stress (100 µg Se L^−1^). Red, blue, brown, and green represent *ChMAT*, *ChMTR*, *ChCOMT*, and *ChSAHH*, respectively. Each data point represents the mean ± standard deviation (SD) (*n* = 3). Error bars represent the standard deviation

**Fig. 11:**
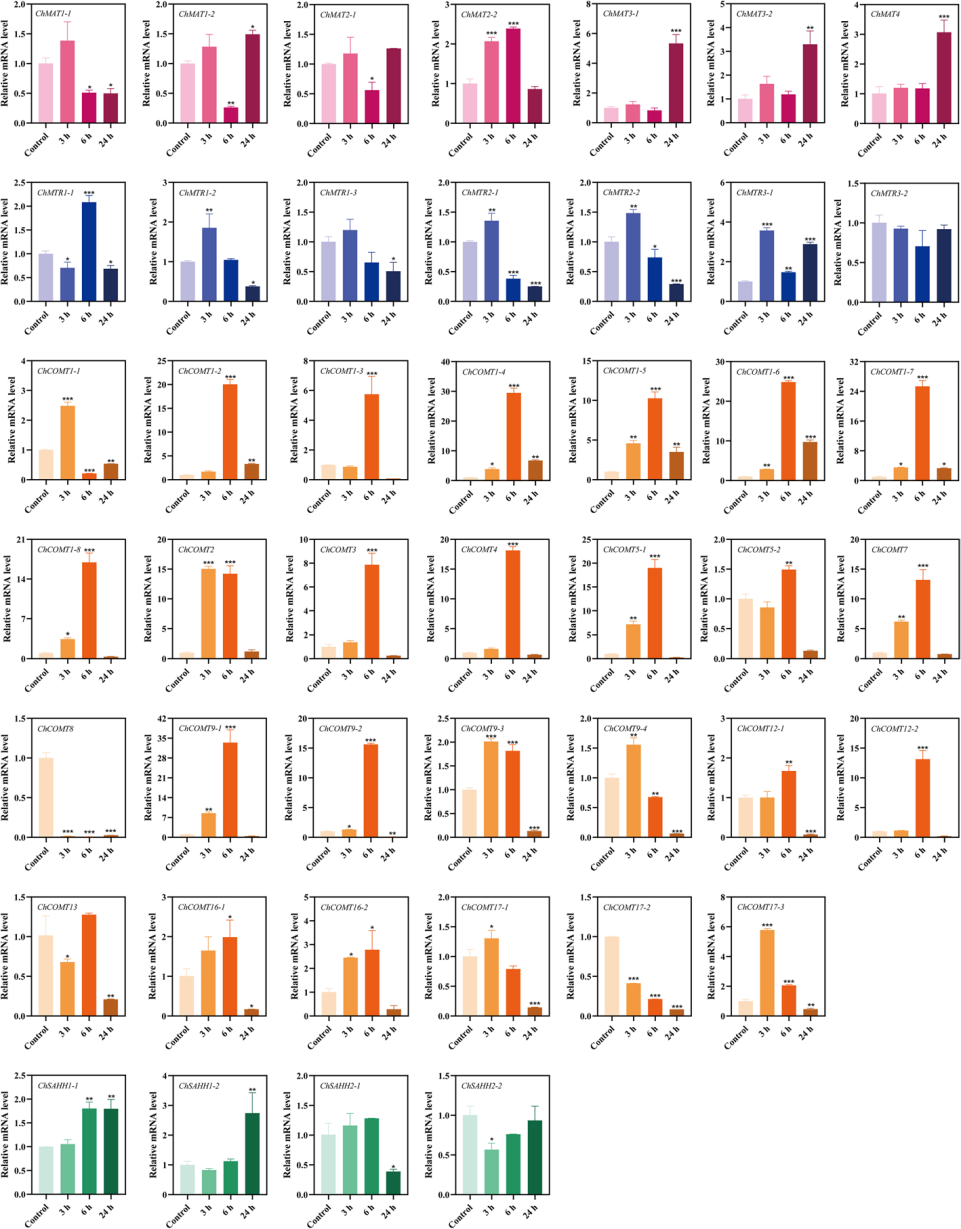
Expression of *ChSeMTC* genes in roots under low-concentration selenium stress (100 µg Se L^−1^). Red, blue, brown, and green represent *ChMAT*, *ChMTR*, *ChCOMT*, and *ChSAHH*, respectively. Each data point represents the mean ± standard deviation (SD) (*n* = 3). Error bars represent the standard deviation

**Fig. 12:**
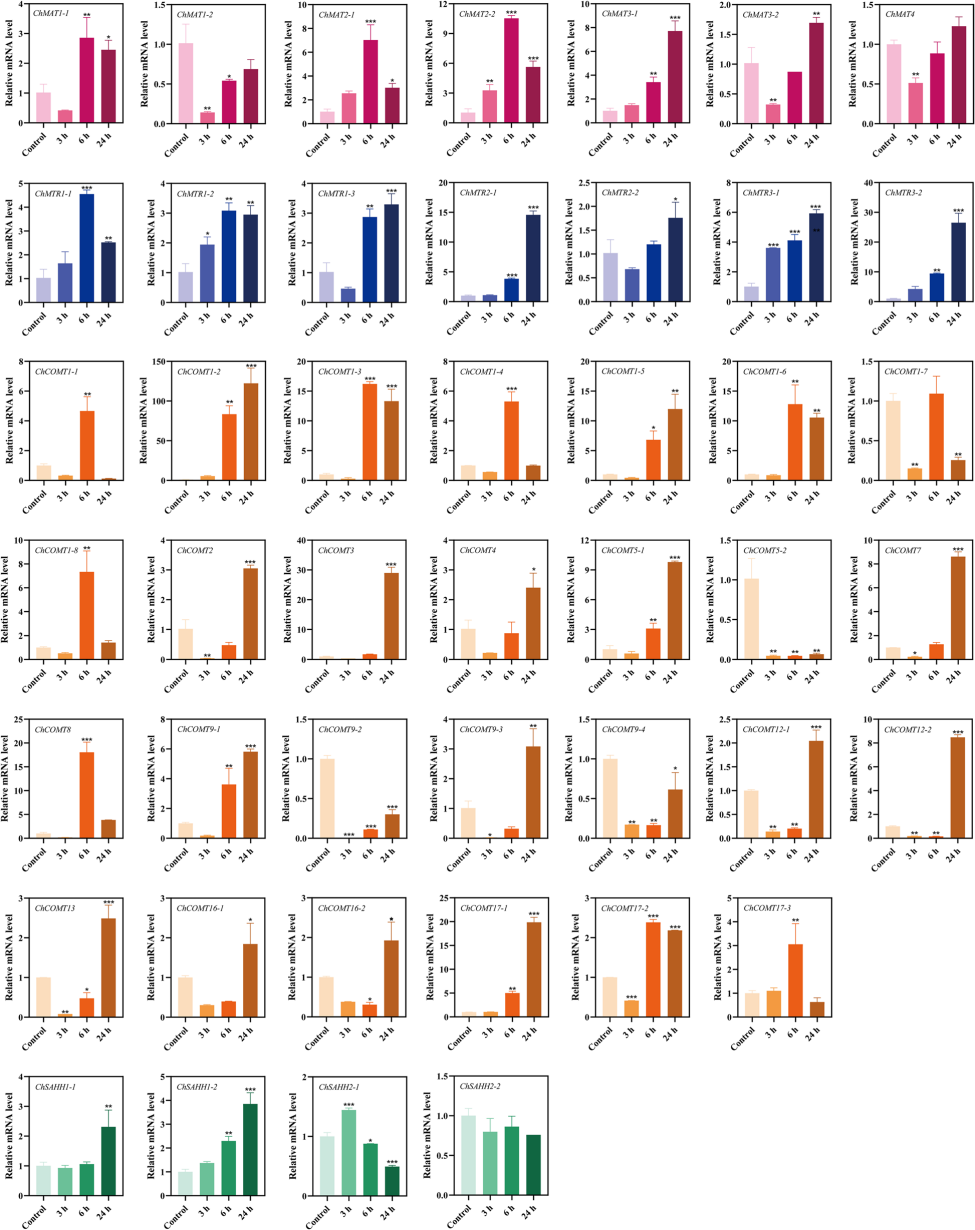
Expression of *ChSeMTC* genes in leaves under high-concentration selenium stress (80,000 µg Se L^−1^). Red, blue, brown, and green represent *ChMAT*, *ChMTR*, *ChCOMT*, and *ChSAHH*, respectively. Each data point represents the mean ± standard deviation (SD) (*n* = 3). Error bars represent the standard deviation

**Fig. 13:**
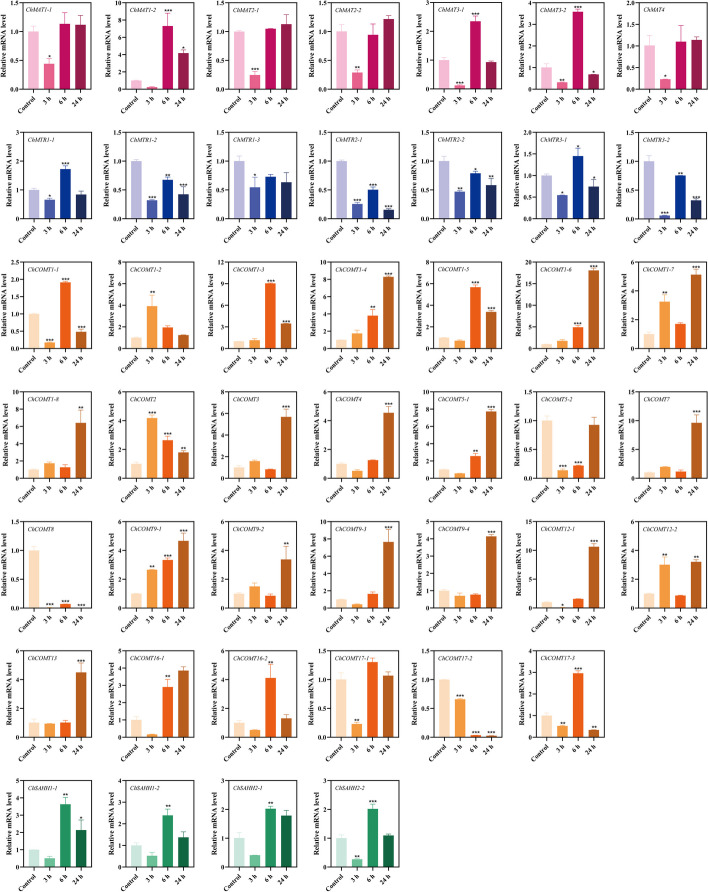
Expression of *ChSeMTC* genes in roots under high-concentration selenium stress (80,000 µg Se L^−1^). Red, blue, brown, and green represent *ChMAT*, *ChMTR*, *ChCOMT*, and *ChSAHH*, respectively. Each data point represents the mean ± standard deviation (SD) (*n* = 3). Error bars represent the standard deviation

RT-qPCR technology was used to further verify the molecular functions of *ChSeMTC* genes under selenium stress to analyze the expression levels in leaves and roots under low-concentration and high-concentration selenium stress. At 24 h after treating the seedlings of *C. hupingshanensis* with 100 µg Se L^−1^ selenite, a significant upregulation of 18-fold was observed in the expression of *ChMAT3-1* genes in the leaves (Fig. 10). Likewise, the expression levels of *ChMAT1-1*, *ChMAT2-1*, and *ChMAT2-2* were also highly upregulated by more than 7-fold at the same time point. In the roots, the expression of *ChMAT3-1* was significantly upregulated by approximately 5.7-fold at 24 h, while *ChMAT3-2* and *ChMAT4* showed an upregulation of approximately 3.6-fold at the same time point (Fig. 11). When the seedlings of *C. hupingshanensis* treating with 80,000 µg Se L^−1^ selenite, a majority of the *ChMAT* members in the leaves exhibited an increase in expression (Fig. 12). *ChMAT2-2* was more significantly upregulated than the other genes, with an upregulation of approximately 10.7-fold at 6 h. *ChMAT2-1* was upregulated approximately 7.9-fold at 6 h and *ChMAT3-1* was upregulated approximately 8.3-fold at 24 h. For the members of the *ChMAT* members in roots, *ChMAT1-2* was significantly upregulated approximately 8.3-fold at 6 h (Fig. 13).

Most members of *ChCOMT* were upregulated in the expression of leaves and roots after treatment with 100 µg Se L^−1^ selenite. *ChCOMT9-1* was highly upregulated approximately 38.7-fold in leaves at 24 h (Figs. 10), 36.3-fold in roots at 6 h (Fig. 11). *ChCOMT1-2*, *ChCOMT1-6*, *ChCOMT5-1*, *ChCOMT7*, *ChCOMT12-2* and *ChCOMT17-3* were upregulated in leaves and roots (Figs. 10 and 11). *ChCOMT3* was more significantly upregulated than the other *ChCOMT* genes, with an upregulation of approximately 30-fold at 24 h in leaves after treatment with 80,000 µg Se L^−1^ selenite (Fig. 12). *ChCOMT1-2*, *ChCOMT1-3*, *ChCOMT1-5*, *ChCOMT1-6*, *ChCOMT5-1* and *ChCOMT17-1* were upregulated in leaves. *ChCOMT1-6* was upregulated approximately 18.4-fold at 24 h in roots (Fig. 13). *ChCOMT7* and *ChCOMT12* were significantly upregulated more than 10-fold at 24 h, and the other genes were upregulated less than 8-fold.

*ChSAHH1-2* was significantly upregulated approximately 11.6-fold, and *ChSAHH1-1* was upregulated approximately 7.6-fold at 24 h in leaves treated with 100 µg Se L^−1^ selenite (Fig. 10). *ChAHH1-2* was upregulated approximately 3.4-fold at 24 h in roots (Fig. 11), *ChSAHH1-2* was upregulated approximately 4.1-fold in leaves (Figs. 12) and 3.3-fold at 6 h in roots (Fig. 13) treated with 80,000 µg Se L^−1^ selenite.

*ChMTR3-2* was highly upregulated approximately 10.7-fold at 24 h in leaves treated with 100 µg Se L^−1^ selenite (Fig. 10). *ChMTR1-2*, *ChMTR2-1* and *ChMTR2-2* were upregulated approximately 5-fold. On the other hand, the expression of *ChMTR3-1* in roots was shown to be highly upregulated at 3 and 12 h, with an upregulation of approximately 3-fold (Fig. 11). The expression of members of the *ChMTR* family genes was upregulated in leaves treated with 80,000 µg Se L^−1^ selenite (Fig. 12). It is worth noting that the *ChMTR2-1* and *ChMTR3-2* genes were significantly upregulated 15- and 28-fold at 24 h. For the members of the *ChMTR* family in roots, *ChMTR1-1* was upregulated approximately 1.8-fold (Fig. 13), and the expression levels of other genes appeared to be downregulated at 3 h, 6 h, 12 h, and 24 h.

## Discussion

In the present study, 45 SeMTC enzymes were identified in *C. hupingshanensis*, comprising 7 *ChMTR*, 7 *ChMAT*, 27 *ChCOMT*, and 4 *ChSAHH* genes. The abundance surpasses that of *Arabidopsis thaliana*, which possesses 26 genes. The most closely related members in the phylogenetic tree exhibited common motif compositions. Through the analysis of conserved domain and multiple sequence, it was determined that all SeMTC proteins in *C. hupingshanensis* contain the conserved domains. Additionally, the results of homologous protein modeling indicated that members of the same family shared similar protein tertiary structure features. These findings suggest that throughout its evolution, *C. hupingshanensis* has developed an increased number of genes to adapt to high selenium environments.

SeMTC is an important part of the metabolism of SeMet, which can lead to selenium atom transfer to sec residues in selenoproteins by a series of enzymes that include MAT, MTase, SAHH, MTR, CγS, and CβL in animals [66, 67]. In this study, the affinity of enzymes of SeMTC with selenium metabolite were analyzed in *C. hupingshanensis*, revealing that SeMTC may also be present in plants with the same pathway in yeast and mammals. By molecular docking analysis, the conserved domains presenting in ChSeMTC constitute the catalytic sites of the enzymes. ChCOMT exhibited a stronger affinity with SeAM compared to sulfur metabolites, and the amino acid residues involved in the interaction is Leu^221/231^ and Gly^207/249^ in catalytic sites. ChSAHH also displayed a stronger affinity with SeAH, but the amino acid residues involved in the interaction is Asn^87^, Asp^139^ and Thr^206/207/208/325^ in maximum affinity binding site. The location of the amino acid residues involved in the interaction at the maximum affinity binding site, rather than the catalytic site, can be attributed to the limitations of computer algorithms employed for molecular docking, which may not fully capture the actual conformation changes of proteins. As a result, when the protein is docked to the substrate, the maximum affinity binding region may not necessarily appear in the catalytic domain. In addition, the affinity of ChMAT with SeMet/Met and ChMTR with SeHcys/Hcys did not exhibit significant differences, suggesting that MAT may not effectively differentiate between Met and SeMet.

Notably, the upregulation extent of most genes under high selenium stress is significantly lower than that under low selenium stress, while *ChCOMT* gene expression remains active under both high and low selenium stress, particularly in leaves. Similar occurrences were also observed in other selenium hyperaccumulators, such as *S. pinnata* and *Cardamine violifolia* [11, 68]. This suggested that numerous methylation reactions occurred in the leaves of *C. hupingshanensis* under selenium stress, particularly those related to lignin synthesis. This is consistent with earlier studies on selenium-treated *C. hupingshanensis* seedlings, which found significant changes in gene expression related to lignin synthesis [17]. SAM is an important methyl donor for the formation of ferulic acid that is a precursor for lignin synthesis, while selenium shares chemical properties with sulfur [23]. This suggested that SeAM may take on some of the roles of SAM, becoming a methyl donor to participate in many transmethylation reactions, such as those in phenylpropane metabolism pathway responsible for lignin synthesis. Moreover, it was found that only *ChMTR* family genes are significantly expressed under high-concentration selenium stress in leaves. These results indicated that a large amount of SeHcys may be involved in the regeneration of SeMet under the stress of high selenium to regulate the balance between SeCys and SeMet for the SeMTC, which maintains a stable level of SeMet and enters the cycle again. This process is similar to animals, a large amount of SeHCys followed the SeMTC to produce SeMet and subsequently entered the methionine pool [67]. Subsequently, SeAM, SeAH, and SeHcys were produced again by the SeMTC [66].

## Conclusion

In summary, 45 genes involved in SeMTC were identified from the *C. hupingshanensis* genome, and the phylogenetic relationships with *A. thaliana* and other closely related species were analyzed. The gene structure, motif composition and homologous protein modeling were analyzed, illustrating that proteins from the same family have similar and conserved sequences. Molecular docking revealed that four enzymes involved in SeMTC have a high affinity for selenium metabolites compared with sulfur. In addition, gene expression levels additionally indicate that SeMTC may also be present in plants, which have equal importance with the methionine cycle under the stress of high selenium. This study predicted the structure, evolution, and expression under Se stress of ChSeMTC, paving the way for future functional analysis of ChSeMTC genes and enhancing our understanding of the physiological and biochemical mechanisms involved in selenium metabolism in plants.

### Supplementary Information

**Additional file 1.** **Fig. S1.** Multiplexed alignment of full sequences of MAT protein in *C. hupingshanensis*; **Fig. S2.** Multiplexed alignment of full sequences of COMT protein in *C. hupingshanensis*; **Fig. S3.** Multiplexed alignment of full sequences of SAHH protein in *C. hupingshanensis*; **Fig. S4.** Multiplexed alignment of full sequences of MTR protein in *C. hupingshanensis*; **Fig. S5.** Predicted 3D structures of ChMAT by the SWISS-MODEL server; **Fig. S6.** Predicted 3D structures of ChCOMT by the SWISS-MODEL server; **Fig. S7.** Predicted 3D structures of ChSAHH by the SWISS-MODEL server; **Fig. S8.** Predicted 3D structures of ChMTR by the SWISS-MODEL server;**Fig. S9.** Interactions of the binary 5-Methyltetrahydrofolate-ChMTR complex with SeHcys; **Fig. S10.** Interactions of the binary ATP-ChMAT complex with SeMet; **Fig. S11.** Interactions of the binary ChCOMT with SeAM; **Fig. S12.** Interactions of the binary ChSAHH with SeAH. 

**Additional file 2.**  **Table S1.** The coding sequences and protein sequences of genes involved in selenomethionine cycle. **Table S2.** The primers of genes involved in selenomethionine cycle for qRT-PCR.

## Data Availability

Data sharing not applicable to this article.
