# Supplementary material for: Whole genome identification, molecular docking and expression analysis of enzymes involved in the selenomethionine cycle in Cardamine hupingshanensis
Source: BMC Plant Biol. 2024 Mar 19;24:199. doi: 10.1186/s12870-024-04898-9 (PMC10949594; doi:10.1186/s12870-024-04898-9)
Supplement: Supplementary file 2 — Additional file 2. Table S1. The coding sequences and protein sequences of genes involved in selenomethionine cycle. Table S2. The primers of genes involved in selenomethionine cycle for qRT-PCR. [file 12870_2024_4898_MOESM2_ESM.docx]

**Supplementary Materials:**


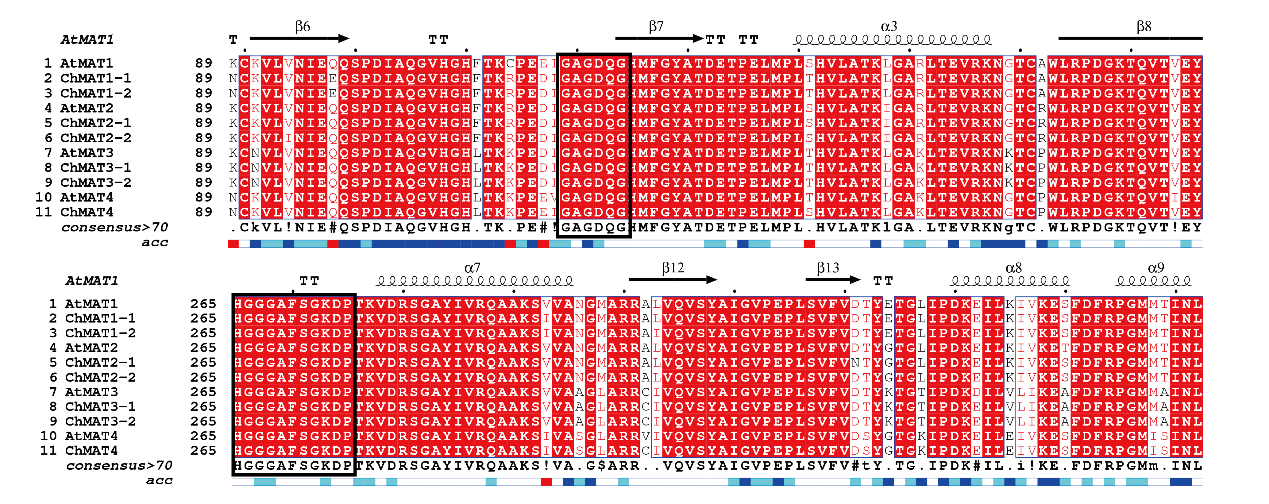
Fig. S1: Multiplexed alignment of full sequences of MAT protein in *C. hupingshanensis*.


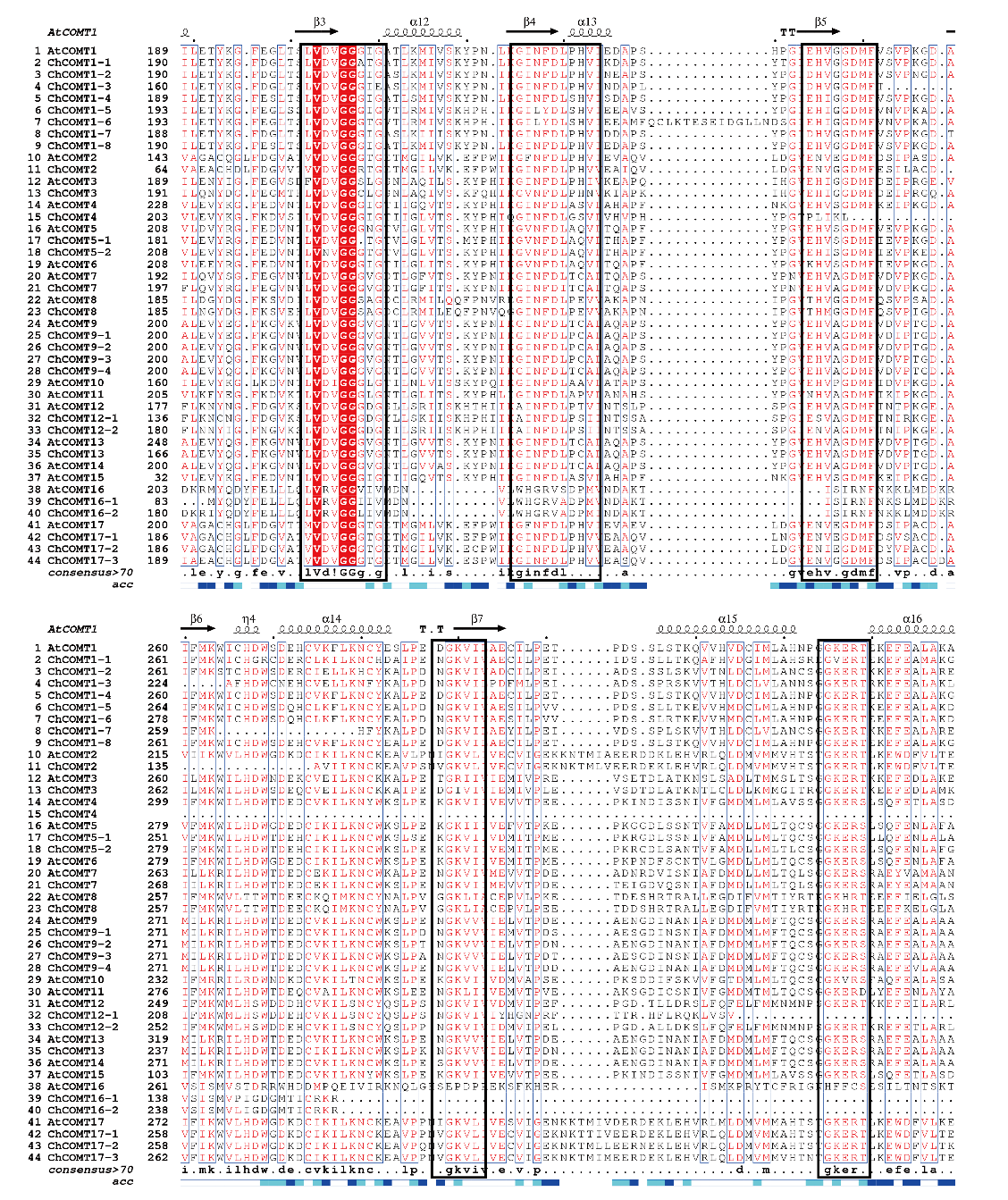


Fig. S2: Multiplexed alignment of full sequences of COMT protein in *C. hupingshanensis*;


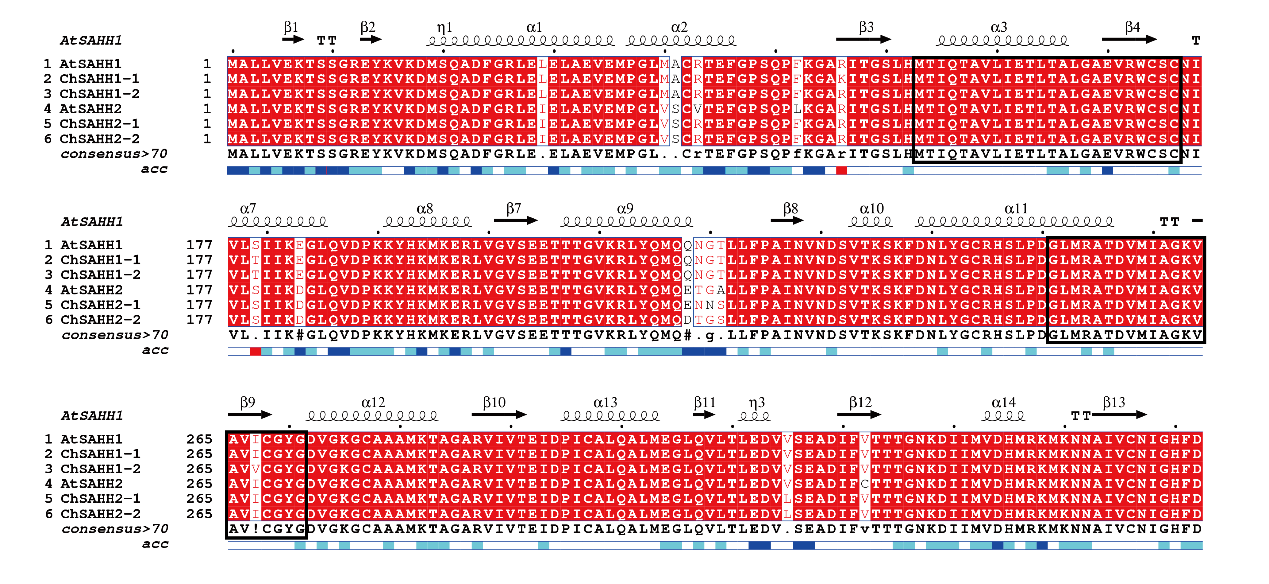


Fig. S3: Multiplexed alignment of full sequences of SAHH protein in *C. hupingshanensis*;


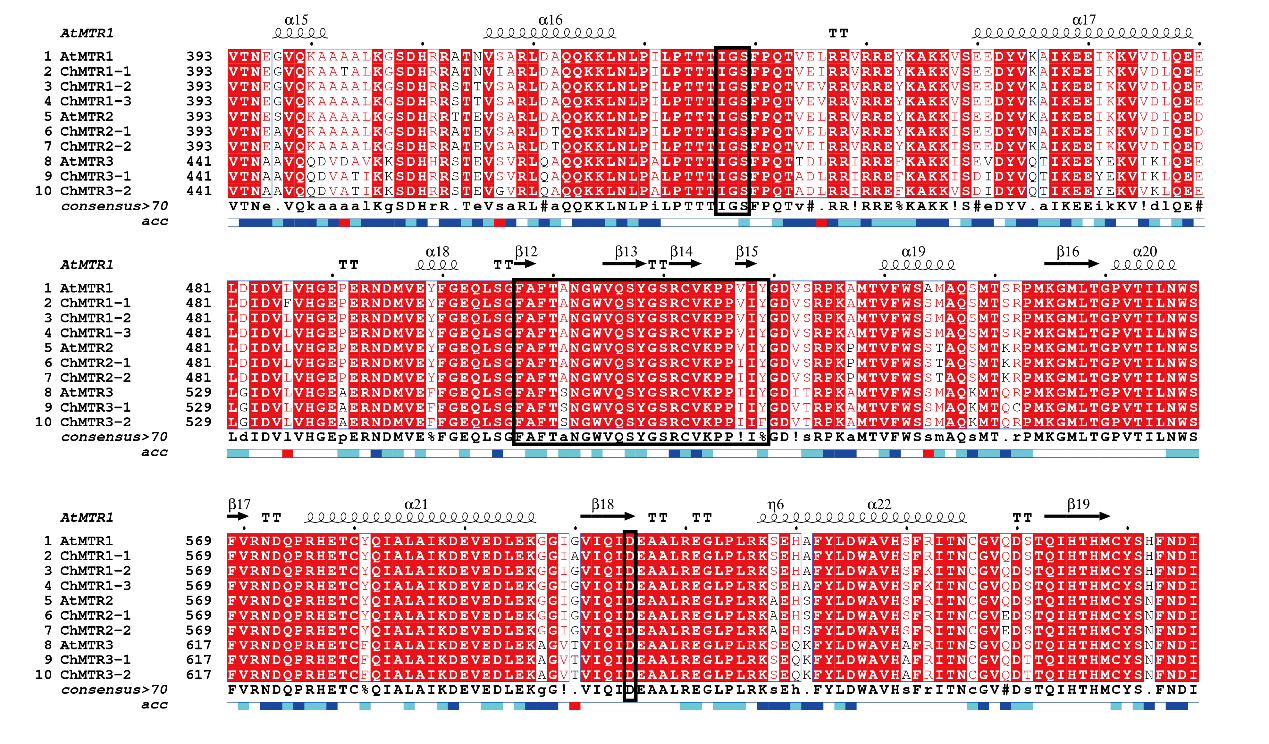


Fig. S4: Multiplexed alignment of full sequences of MTR protein in *C. hupingshanensis*;


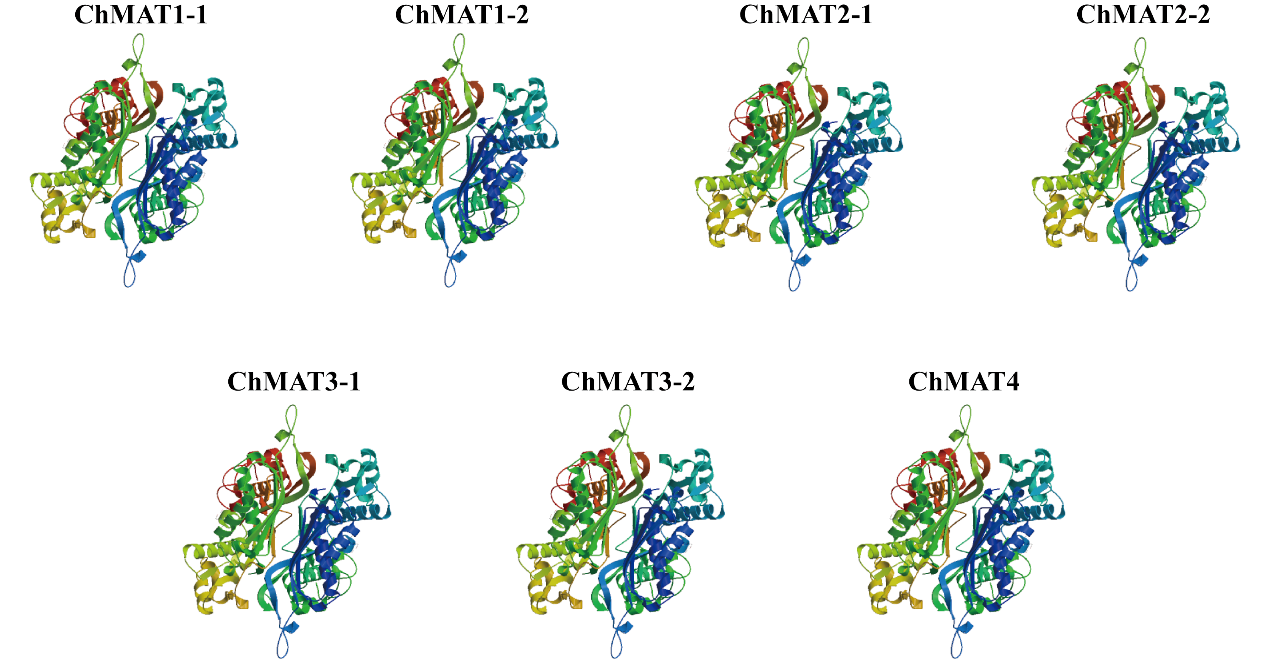


Fig. S5: Predicted 3D structures of ChMAT by the SWISS-MODEL server;


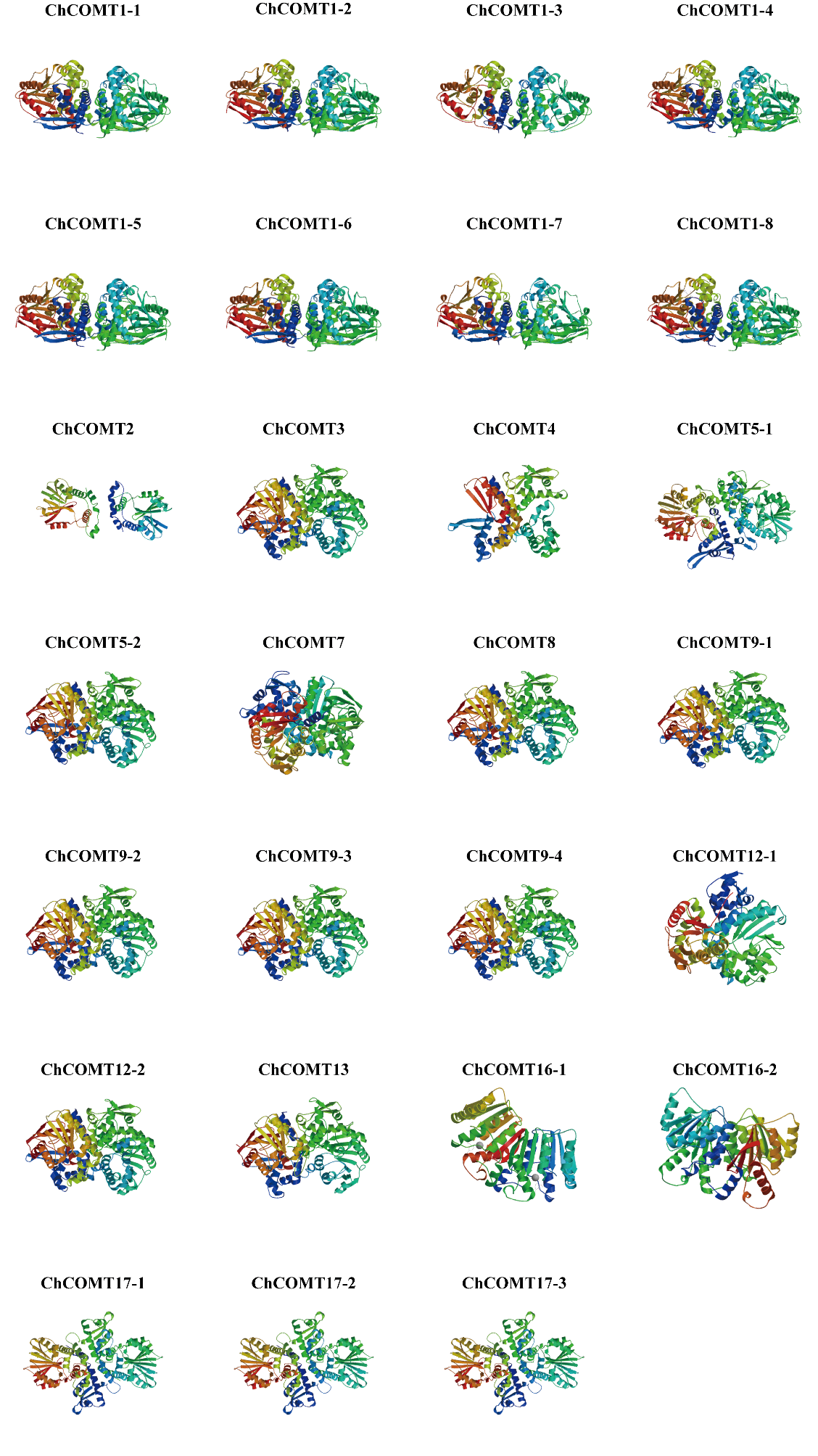


Fig. S6: Predicted 3D structures of ChCOMT by the SWISS-MODEL server;


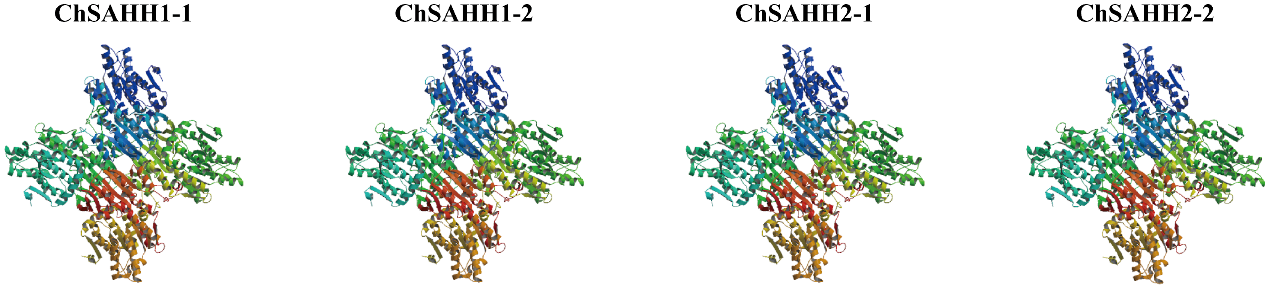


Fig. S7: Predicted 3D structures of ChSAHH by the SWISS-MODEL server;


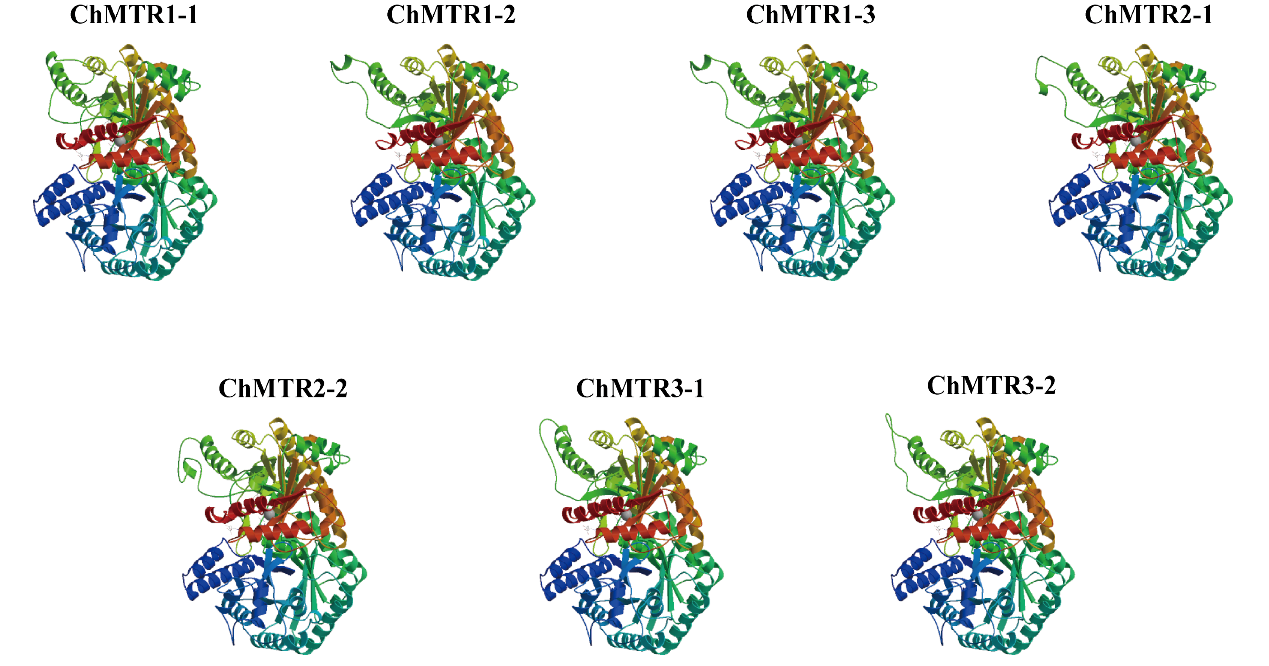


Fig. S8: Predicted 3D structures of ChMTR by the SWISS-MODEL server;


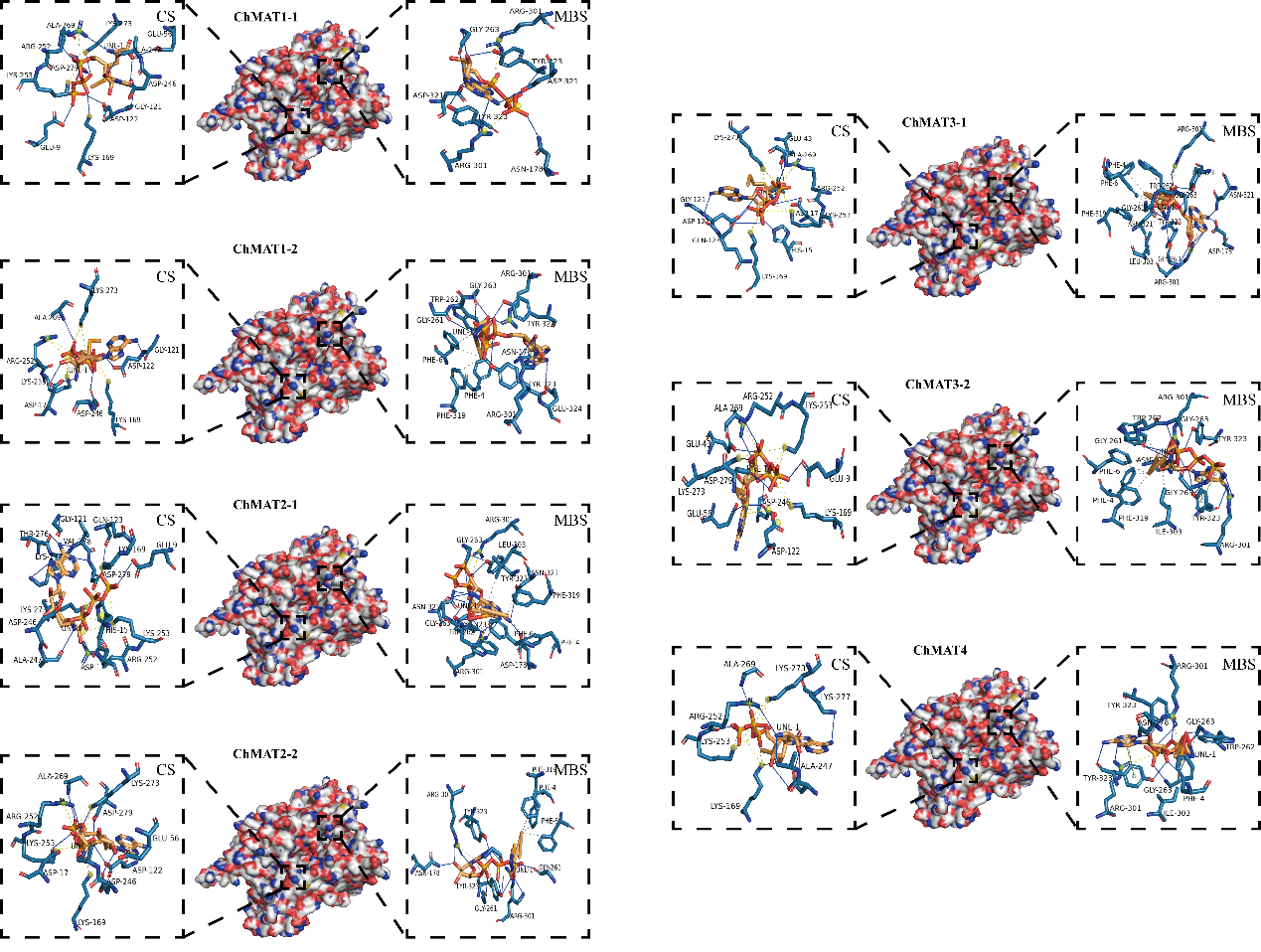


Fig. S9: Interactions of the binary 5-Methyltetrahydrofolate-ChMTR complex with SeHcys;


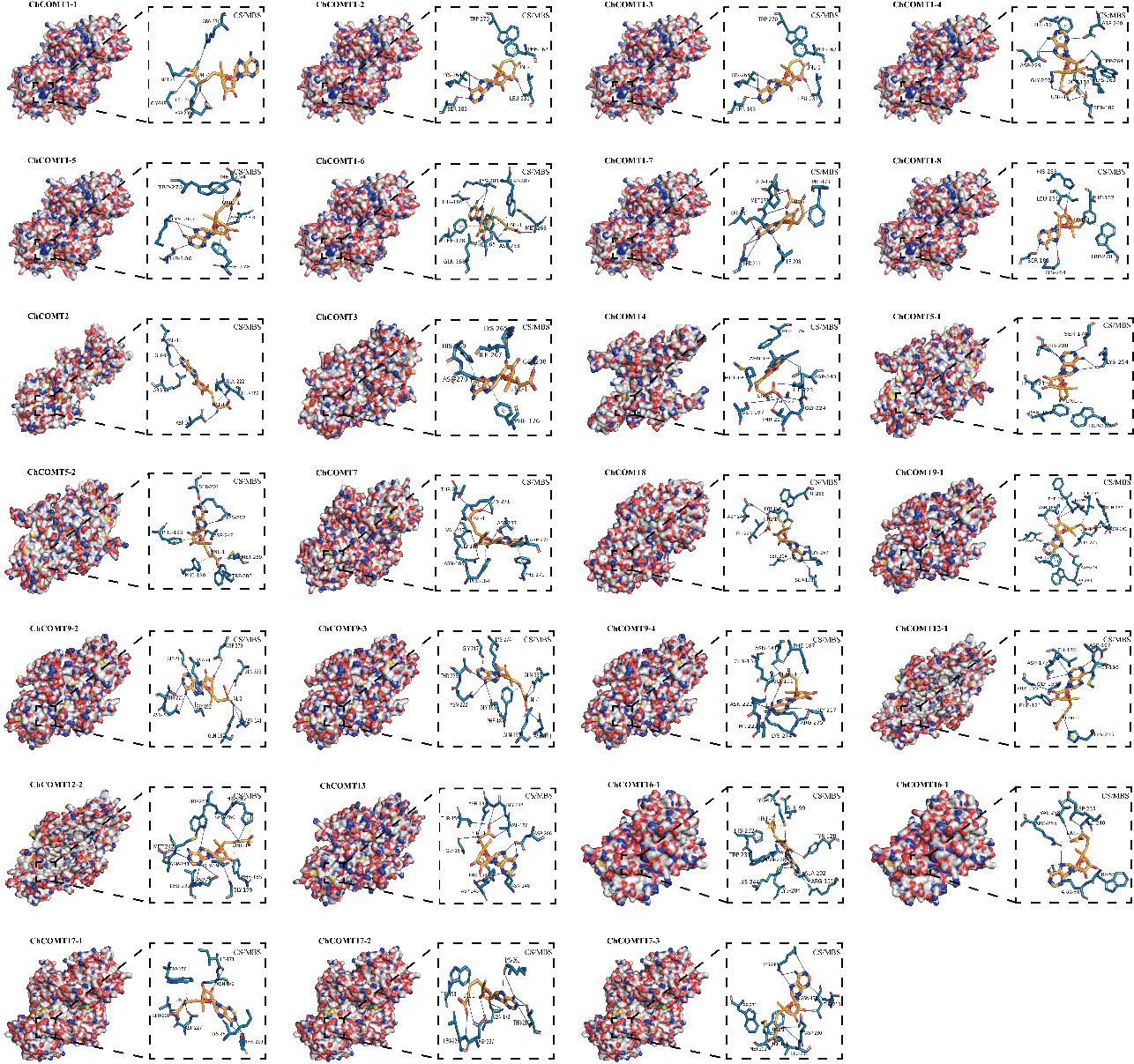


Fig. S10: Interactions of the binary ATP-ChMAT complex with SeMet;


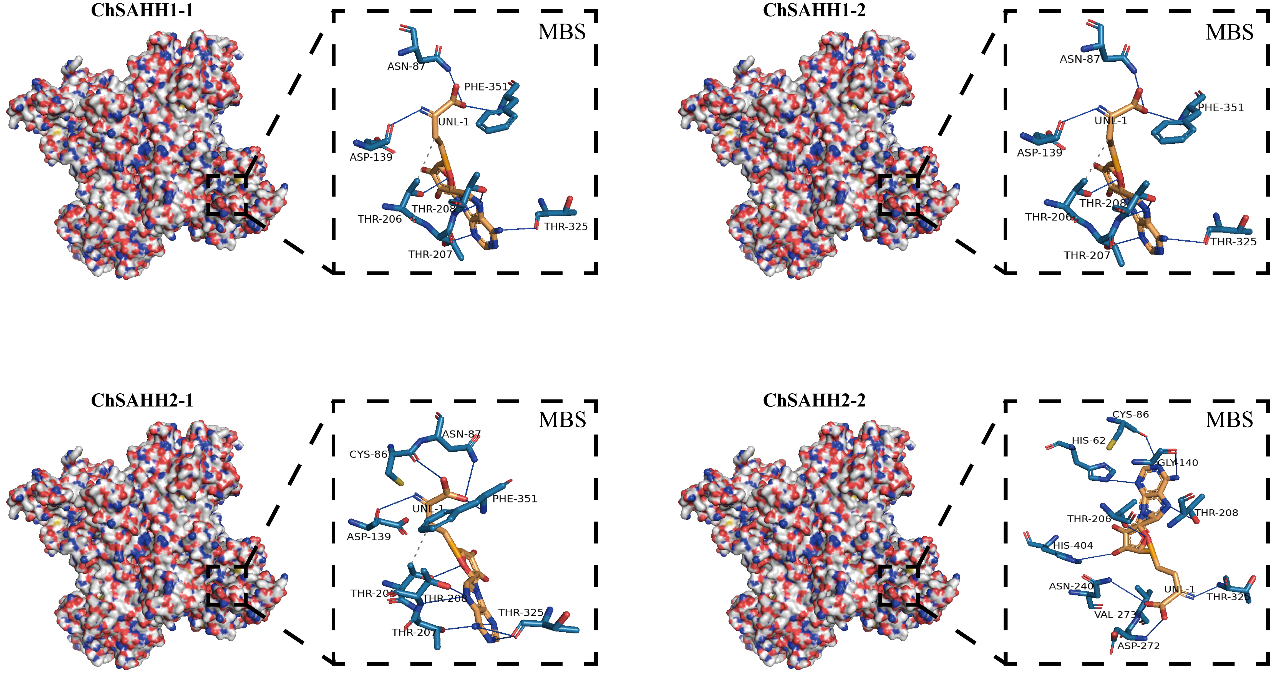


Fig. S11: Interactions of the binary ChCOMT with SeAM;


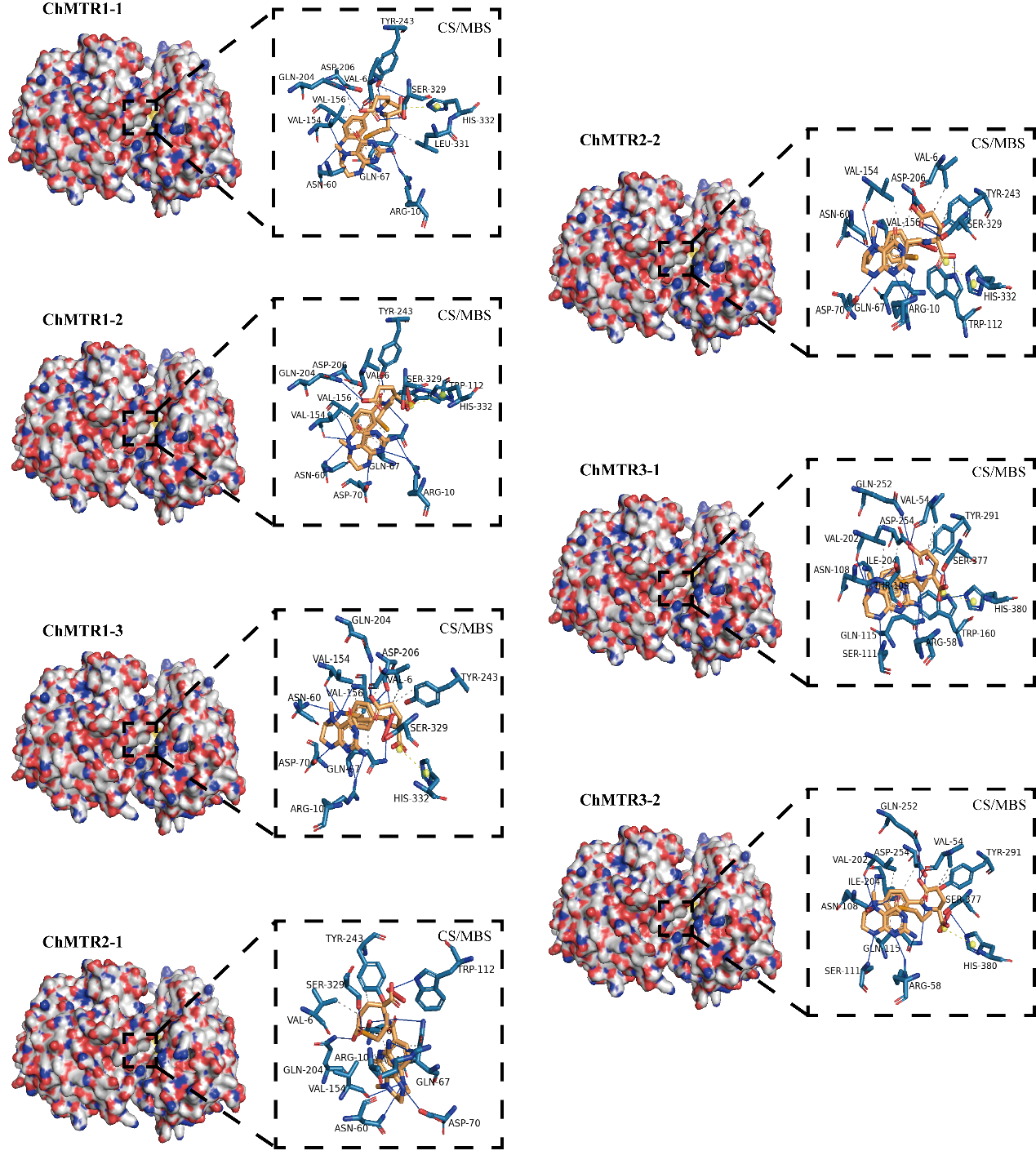


Fig. S12: Interactions of the binary ChSAHH with SeAH;
